# Small-nucleolar RNA host gene3 (SNHG3) and leukemia-associated non-coding IGF1R activator RNA 1 (LUNAR1) correlated with CRC patients’ clinical features: a step-toward ncRNA-precision

**DOI:** 10.1038/s41598-026-37432-y

**Published:** 2026-02-26

**Authors:** Omnia Emam, Eman F. Wasfey, Mostafa Elnakib, Mohamed Yassin, Sherif Abdel Halim, Nadia M. Hamdy

**Affiliations:** 1 Egyptian Drug Authority, Cairo, Egypt; 2 Biochemistry and Molecular Biology Department, Faculty of Pharmacy, Ain Shams University, Abassia, Cairo, 11566 Egypt; 3 Department of Medical Microbiology & Immunology, Military Institute of Health and Epidemiology, Military Medical Academy, Cairo, Egypt; 4 Armed Forces Laboratories Medical Research and Blood Bank, Military Medical Academy, Cairo, Egypt; 5 Clinical Oncology Department, Faculty of Medicine, Ain Shams University, Abassia, Cairo, 11566 Egypt; 6 Department of General and Colorectal Surgery, Faculty of Medicine, Ain Shams University, Abassia, Cairo, 11566 Egypt

**Keywords:** Notch, SNHG3, LUNAR1, lncRNAs, Colorectal cancer, Prognosis, Epigenetics, Inflammation, Hallmarks of cancer, In silico/Bioinformatics analysis, Biomarkers, Cancer, Computational biology and bioinformatics, Genetics, Oncology

## Abstract

Notch-signaling is implicated in tumorigenesis as well as Notch-associated long non-coding RNAs (lncRNAs) small nucleolar RNA host gene3 (SNHG3) and leukemia-associated non-coding IGF1R activator RNA 1 (LUNAR1) are highly expressed in various malignant tissues including colorectal cancer (CRC). However, the clinical and prognostic utility of these lncRNAs in CRC patients’ blood is still lacking. The aim of this study was to assess the expression patterns of circulatory Notch-associated lncRNAs SNHG3 and LUNAR1 and to explore their relevance for CRC follow up and risk assessment. Subject and methods: Quantitative real-time polymerase chain reaction (qRT-PCR) was used to quantify the expression levels of SNHG3 and LUNAR1 in sera of 70 Egyptian CRC patients’ and to compare them with 26 age- and sex-matched apparently healthy volunteer control group. Results: Serum fold-change expression of SNHG3 and LUNAR1 were up-regulated in CRC patients compared to the apparently healthy controls (*p* < 0.0001). SNHG3 lncRNA fold expression levels were positively correlated with advanced CRC stage (III-IV) (*p* = 0.0175) being highly related to poorer clinicopathological features of CRC patients including extensive tumor invasion (*p* = 0.0046), vascular invasion *(p* = 0.0015), and lymph node metastasis (*p* = 0.0175). Likewise, LUNAR1 lncRNA fold-expression level was significantly positively associated with larger tumor size *(p* = 0.0033) and deeper tumor invasion (*p* = 0.042). The two lncRNAs had higher discriminative utility than either CEA or CA19-9 per the receiver operating characteristic (ROC) curve, with higher sensitivities and specificities (*p* < 0.0001). A significant positive correlation between SNHG3 and LUNAR1 lncRNAs fold expressions (r = 0.4615, *p* < 0.0001). Conclusion: SNHG3 and LUNAR1 might be useful non-invasive bio-molecular markers for CRC monitoring. Being correlated with poorer CRC patients’ clinical features, SNHG3 and LUNAR1 lncRNAs might constitute potential putative therapeutic targets for CRC, per identified downstream genes or proteins in silico, a step toward ncRNA-based precision medicine.

**Supplementary Information:**

The online version contains supplementary material available at 10.1038/s41598-026-37432-y.

## Introduction

Background. Colorectal cancer (CRC) has been identified as a major public health concern given its high incidence and mortality rates^1^. In 2020, CRC was the third-most prevalent malignancy after breast and lung cancer, accounting for 10% of new cases and ranking second in terms of mortality with 9.4% of deaths^2^. Unfortunately, by year 2030, more than 2.2 million new cases and 1.1 million fatalities from CRC are globally anticipated^3^.

Problem. Despite significant improvements in CRC surgical or radiological interventional treatment approaches with neo-adjuvant therapies, patients’ prognosis remains bleak^4,5^. Metastases and post-surgical tumor recurrence are prevalent, particularly, in more advanced cancer cases^6^, which may account for the increased number of cases and fatalities prediction, which undermines national efforts for achieving Egypt Vision 2030 implementing SDGs.

Problem statement. Conventionally used CRC prognostic markers for monitoring treatment outcome and pointing to cancer recurrence are carbohydrate antigen 19.9 (CA19.9) and/or carcinoembryonic antigen (CEA) that are not that sensitive^7^ per patients subclassification or stratification related to CRC pathological characteristics for more efficient and earlier prognosis. Thence, a growing demand for sensitive and precise bio-molecular marker(s) that correlate better with CRC prognostic markers and/or CRC clinical outcome^8^, that is if successful would constitute the bases for “Better Health” SDG#3 and less cancer recurrence and, therefore, less mortality.

Liquid biopsies are used for cancer diagnosis or prognosis, like breast cancer, leukemia, liver, bladder cancer or CRC and more diseases, via measurement of tumor-derived bio-molecular markers including circulating ncRNAs including long non-coding RNAs (lncRNAs), microRNA, exosomal ncRNAs, oncogenes or tumor suppressor genes, and their mutations, tumor-related-cytokines, and down-stream target proteins^9–22^.

After extensive literature search and mining together with bioinformatics analysis to mind-the-research-gap(s) for better “Cancer Epigenetics Precision” we have chosen two notch-related lncRNAs to study in relation-to-CRC prognosis. A hot area of research, nowadays, is to figure out the impact of Notch-related ncRNAs on CRC risk and/or progression.

Notch-signaling pathway is ubiquitous cascade within species to control a broad variety of biological events, including cell division, proliferation, and cell death^23,24^. Recent investigations have revealed the fundamental role of Notch-cascade in CRC evolution^25^. Intestinal epithelial cells’ homeostatic self-renewal and tumor-promoting transformation can both be managed by Notch signaling^26^. Stimulation of Notch-cascade can be epigenetically triggered by dysregulated non-protein coding RNAs’ (ncRNAs) expressions^27^.

The significant utility of tumor-expressed Notch-associated lncRNAs as prognostic malignancy indicators proves how they are connected to carcinogenesis or metastasis and therefore, reflect the outcome(s) in different cancer types including CRC^28–31^.

Small Nucleolar RNA Host Gene3 (SNHG3) is 4950 bp lncRNA situated on chromosome 1p35.3^32^. In breast cancer, upregulated SNHG3 triggers Notch system activation as a result of its competitive binding to human homo sapiens (hsa) micro-RNA (miR) hsa-miR-154-3p exacerbating proliferation and metastasis of cancer cells^33^. Moreover, SNHG3 positively regulates Notch1 expression in ovarian cancer through hsa-miR-139-5p suppression, accelerating tumor cells’ proliferation and migration^34^. SNHG3 exerted a carcinogenic role in prostate cancer, osteosarcoma, glioma, gastric cancer, laryngeal cancer, bladder cancer, and CRC^32^. Huang et al. reported SNHG3 elevated expression in CRC cells and tissues, stimulating cancer progression through sponging hsa-miR-182-5p^35^. Therefore, SNHG3 is considered as a malignancy enhancer that regulates Notch system in various cancer types.

Leukemia-associated nc-insulin-like growth factor1 receptor (IGF1R)-Activator RNA1 (LUNAR1) serves as a downstream target of Notch-signaling, at the same time LUNAR1 acts through Notch-signaling stimulation. LUNAR1 is a transcript of 491 nucleotides (nt) gene on chromosome 15q26.3 with four exons and poly (A) tail^36^. LUNAR1 was identified to be elevated in CRC tissues, triggered by Notch1 stimulation, accelerating CRC progression via retaining IGF1R expression^37^, being a positive regulator of cell division.

Aim of the study: Per, few research publications on both notch-related lncRNAs, SNHG3 and LUNAR1, in CRC prognosis or CRC risk assessment as well as patients’ stratification based on clinico-pathological characteristics, therefore, assessment of the clinical utility of SNHG3 and LUNAR1 lncRNAs fold change expressions in CRC patients’ peripheral blood liquid biopsy (the easy non-invasive sample) is important as step toward implementing ncRNA precision.

Study objective(s) to assess, first, the expression level and pattern of Notch-associated lncRNAs SNHG3 and LUNAR1 in peripheral blood liquid biopsy, collected from treatment-naïve Egyptian CRC patients’ cohort, compared to age-matched and sex-matched apparently healthy volunteer subjects as controls. Second, to evaluate lncRNAs SNHG3 and LUNAR1 expression usefulness as sensitive, non-invasive prognostic bio-molecular marker(s) for CRC screening. Third, to investigate the correlation between SNHG3 and LUNAR1 expression with CRC clinic-pathological features. Finally, to explore the clinical relevance of the investigated lncRNAs for CRC patients’ clinical features outcomes assessment. All these objectives to be confirmed or ruled out by in silico analysis and bioinformatics databases search.

## Subjects and methods

### Sample size and power of the study

In accordance with prior reference studies^36,38^ the sample size was estimated utilizing sample size online calculator https://riskcalc.org/samplesize/# for comparison of the area under the curve (AUC) with a null hypothesis value (done January, 2021) through using two-sided significance level of 0.05 and the power (1-beta) of 0.95 as the two-sided confidence level of 95%. AUC of SNHG3 from the reference paper = 0.863, ratio of positive cases/total sample size = 0.73. AUC of LUNAR1 from the reference paper = 0.942, ratio of positive case /total sample size = 87/115 = 0.756. These AUCs from such a probability (power) the sample size is 62 for SNHG3 and 64 for LUNAR1. Per, to compare disease samples to control as 2:1, so groups will be 45 samples for CRC patients and 17 controls for SNHG3 and 48 CRC patients’ vs 16 controls for LUNAR1.

### Study design

Case-controlled, pilot, prospective observational exploratory study. The study was carried out from June, 2021 to October, 2022.

### Study participants

#### Patients’ group

70 CRC Egyptian patients, admitted to the Oncology Center of the Faculty of Medicine, Ain Shams University Hospitals (ASUH) or the Oncological Surgery Department of Dar-El-Shafa Hospital, before surgical treatment or CRC neo-adjuvant treatment, if they were eligible and agreed to participate (signed the I.C) were recruited in the research. Male-to-female was 30:40 with an age-range; minimum–maximum 24–79 years.

#### Apparently healthy control group

26 randomly-chosen, age-matched, and sex-matched healthy subjects served as the control group. Male-to-female was 9:17 and age range; minimum–maximum 35–78 years. Control group subjects were chosen from those who came to visit hospital workers in the hospital or from blood donors at the ASUH Blood Donation Unit. None of the control group did take any medications or have any diseases upon questioner and blood samples was taken for CBC.

#### Inclusion and exclusion criteria

Patients, came to the Oncology Center of ASUH or the Oncological Surgery Department of Dar-El-Shafa Hospital, who experienced a range of colonic symptoms, such as constipation, abdominal discomfort, rectal bleeding, and abrupt weight loss were included in the study when diagnosis of CRC was clinically confirmed by abdominal radiography, colonoscopy, and histopathology.

Exclusion criteria patients who received chemotherapy, radiotherapy or undergone surgery. Patients with other types of cancer were also excluded. Individuals with missing data were not included.

#### Patients pathological and clinical data

For each CRC participant’s colonoscopy outcomes, abdominal radiographic imaging, pathological evaluations were used to define CRC staging. Tumor lymph-node metastasis (TNM) staging criteria was based on the American Joint Committee on Cancer (AJCC)^39^ criterion.

CRC study participants’ family history, hypertension (HTN) or diabetes mellitus (DM) were recorded as non-communicable diseases (NCD), to correlate them to notch-related lncRNAs studied.

Inflammatory conditions such as ulcerative colitis, tumor size, tumor location (rectal, colonic, or rectosigmoid), mucinous or non-mucinous type, tumor invasion depth, lymph-node metastasis (LNM), presence of vascular invasion or not, tumor differentiation status; adenocarcinoma, moderately differentiated adenocarcinoma or poorly differentiated adenocarcinoma as well as the presence of the sub histologic features, signet ring cell, or not, were collected from eligible volunteering CRC patients’ files.

### In silico analysis

#### Notch-related lncRNA Bioinformatics from various databases via (accessed January, 2021 and revised July, 2023)

Gene Set Enrichment Analysis (GSEA)^40^ with ClusterProfiler utilizing the Kyoto Encyclopedia of Genes and Genomes (KEGG)^41,42^ from Genome net https://www.genome.jp/ was used to analyze the functional enrichment of genes, diseases, networks, drugs, and pathways related to Notch-signaling. Ensemble database search^43^ https://www.ensembl.org/index.html for the potential probable Notch-related lncRNAs *SNHG3* and *LUNAR1* genes National Center for Biotechnology Information (NCBI) https://www.ncbi.nlm.nih.gov/ search for characterization of the investigated hsa lncRNAs *SNHG3* gene and transcripts^2^ and hsa lncRNAs *LUNAR1* gene and transcript^1^. HUGO Gene Nomenclature Committee (HGNC)^44^ https://www.genenames.org/ hsa lncRNAs genes *SNHG3* and *LUNAR1.*

#### LncRNADisease v3.0 expression

LncRNA and Disease Database (version 3.0)^45^ to explore the Notch-related lncRNAs expression CRC retrieved from validated experimental results in publications or predicted http://www.rnanut.net/lncrnadisease/index.php/home

2.2.4.3. Expression via ENCORI Project Pan-Cancer Analysis Platform^46^ https://rnasysu.com/encori/panGeneDiffExp.php of lncRNA or genes across 32 types of Cancers. The expression box-plot values of genes from RNA-seq data were scaled with log2(FPKM + 0.01), while the ones from miRNA-seq data were scaled with log2(RPM + 0.01). Differential expression Analysis for SNHG3, LUNAR1 and its transcript IGF1R expression levels in CRC tumor samples vs control samples.

#### Functional enrichment analysis and targeted pathways

KEGG Targeted Pathways and STRING Protein–Protein Interaction (PPI) Networks version 11.5 https://string-db.org/^47,48^ (Accessed on July, 2023).

LncRNAWiki 2.0 LncRNA—LncRNAWiki—CNCB-NGDC https://ngdc.cncb.ac.cn/lncrnawiki/ (Accessed August 30th, 2023).

### Blood samples

From CRC patients and healthy volunteers, five milliliters of peripheral venous blood fluid biopsy were collected and stored in clot-activating polymer gel vacutainers (Greiner Bio-One GmbH, Australia). The samples were centrifuged in vacutainers at a speed of 4000 rpm for ten minutes at room temperature (25 °C). The obtained serum was aliquoted and stored at − 80 °C in RNAse-free Eppendorf tubes.

#### Total RNA extraction

Using the miRNeasy Mini kit (Cat. No. 217004; Qiagen, Hilden, Germany), total RNA was isolated from sera in accordance with the protocol’s instructions. Aliquots of the extracted RNA were kept at -80 °C after being dissolved in 30 μl of RNase-free water.

#### Quantitation of purified RNA

Using a NanoDrop®-1000 spectrophotometer (Thermo Scientific, Wilmington, DE, USA), the isolated RNA’s purity and concentration are evaluated. RNA’s quantity was determined in the sample utilizing absorbance at 260 nm (A_260_ = 1 → 44 ng/μl). In addition, the purity of RNA was evaluated utilizing A260/280 nm ratios. The acceptable 260/280 ratio ranges from 1.8 to 2.1, whereas the 260/230 ratio is more than 1.7.

#### Reverse transcription

Complementary DNA (cDNA) synthesis was carried out using the High-Capacity cDNA Reverse Transcription Kit with RNase Inhibitor (Cat. No 4374966, Applied Biosystems, ThermoFisher Scientific, USA) according to the manufacturer’s regulations. In a 20 µl reaction volume, reverse transcription was carried out at 25 °C for 10 min, 37 °C for 120 min, and then underwent heat inactivation for 5 min at 85 °C. The produced cDNA was maintained at -80 °C till further investigations.

#### Expression measurement of lncRNAs using quantitative real-time reverse transcription polymerase chain reaction (qRT-PCR)

QRT-PCR was carried out in a 20 µl reaction using the TaqMan® Gene Expression Master Mix (Cat. No 4370048, Applied Biosystems, ThermoFisher Scientific, USA). In order to determine the expression levels of lncRNAs SNHG3 and LUNAR1, TaqMan gene expression assays for human SNHG3 (Hs05055352_s1, Cat. No 4448892, ThermoFisher Scientific, USA) and human LUNAR1 (Hs03829521_s1, Cat. No 4426961, ThermoFisher Scientific, USA) were used and the TaqMan gene expression assay for human glyceraldehyde 3-phosphate dehydrogenase (GAPDH) (Cat. No 4326317E, ThermoFisher Scientific, USA) was used as an endogenous standard to normalize the values. The reaction was conducted using the StepOne™ qRT-PCR technique (Applied Biosystems, CA, USA). The thermal cycling strategy was as follows: a stage of initial uracil-N-glycosylase (UNG) incubation at 50 °C for 2 min, followed by activation step lasting 10 min at 95 °C proceeded by 40 cycles of denaturation for 15 s at 95 °C, annealing and extending for 1 min at 60 °C.

Per the manufacturer, the assay “limit of detection” (LoD) can detect cDNA template from 1 pg to 100 ng in nuclease free water.

The cycle threshold (Ct) technique as a fold change (2^−ΔΔCt^) was used to calculate and normalize the lncRNAs expression levels, utilizing GAPDH as the housekeeping gene. ΔCt was obtained by subtracting the Ct values of GAPDH from either SNHG3 or LUNAR1 Ct values^49^, where; ΔΔCt = ΔCt_CRC samples_ – ΔCt_healthy control samples_.

#### CEA and CA19-9 determination by electrochemiluminescence immunoassay

One portion of the obtained sera was utilized for the purpose of determining CEA and CA19-9 tumor markers. Electrochemiluminescence immunoassay utilizing Cobas® e 602 developed by Roche Diagnostics, GmbH, Germany was used to determine the serum concentrations of CEA (Cat. No 11731629 322) and CA19.9 (Cat. No 11776193500), according to the manufacturer’s protocol.

#### Routine biochemical testing results record from patients’ files

Liver function tests; aspartate aminotransferase (AST) and Alanine aminotransferase (ALT) as well as serum creatinine and serum urea levels, hemoglobin (Hgb), platelet count, lymphocytes count, and prothrombin time (PT) were all gathered from patients’ files.

#### Ratios and indices

In meters, participants’ heights and in kilograms their weights were recorded, to calculate Body mass index (BMI in kg/m^2^) where overweight subjects have BMI of 25–29.9 kg/m^2^, 30 kg/m^2^ or over are obese, while 18.5–24.9 kg/m^2^ is indicative of normal weight,

https://www.nhlbi.nih.gov/health/educational/lose_wt/BMI/bmicalc.htm

The immune response-related inflammation indicator biomarker able to predict the accompanying inflammatory disorder severity is the platelet-to-lymphocytes ratio (PLR)^10,50^.

### Statistical analysis

With the aid of GraphPad Prism® version 9.01 (GraphPad Software, San Diego, CA, USA, SPSS 23.0 (statistical package for social studies software) (IBM, Armonk, NY), MedCalc Statistic Software version 19.1 (MedCalc Software by Ostend, Belgium), and Microsoft Office Excel 2019, statistical analysis was carried out.

Chi-square test (χ^2^) was utilized to assess the associations between participants’ characteristics and the groups. The Shapiro–Wilk normality test as well as Kolmogorov–Smirnov test were applied to determine the pattern of the normal distribution for the both groups and subgroups of data. Data was expressed as mean ± SD when it passed the normality test. Student’s (*t*) test was utilized to determine any significant differences between two normally distributed groups. Additionally, one-way ANOVA (F) followed by post hoc Tukey’s multiple comparison test was used, when required, to determine significant variations between multiple groups. While, the data that didn’t pass the normality test was expressed as median (interquartile range) (IQR) (25th percentile-75th percentile). The Mann–Whitney (U) test was used to pinpoint the significant changes between two sets of participants. Moreover, the Kruskal–Wallis (H) test and consequently Dunn’s multiple comparison test were used, when needed, to determine whether there were statistically significant differences between different groups.

The receiver operating characteristic (ROC) curve as well as AUC were implemented to evaluate the abilities of serum lncRNAs SNHG3 and LUNAR1 for discrimination between groups and with groups for subgrouping identification. The best cut-off, sensitivities (SNs), specificities (SPs) as well as negative and positive predictive values NPVs and PPVs, respectively, were all determined using the ROC curve, with AUC estimated range from 0 to 1.

Negative likelihood ratios (LRs) are employed in medical testing to evaluate the marker discriminative efficiency. The ratio, which denotes the likelihood that a person has the disease or condition, confirms the SNs and SPs identified by the ROC curve. SN and SP provide a different meaning for the LR, with negative LR equal to (100-SN)/SP.

Multiple regression models were used to assess the influence of the participants demographic and patients’ clinicopathological data (as independent factors) on the expression levels of the lncRNAs SNHG3 and LUNAR1 that we considered as the dependent variables (Bonferroni corrections used to control Type I errors). Correlation between numerous variables was assessed using Spearman’s correlation coefficient (*r*). Statistical analysis tests are set significant when the two-tailed* p*-value is < 0.05 (*).

## Results

### In silico analysis

SNHG3 and LUNAR1 genomic regions and chromosomal location and gene product (Table 1) retrieved from HGNC; HUGO Gene Nomenclature Committee https://www.genenames.org/, NCBI; National Center for Biotechnology Information https://www.ncbi.nlm.nih.gov/ and Ensembl https://www.ensembl.org/index.html bioinformatics databases (Accessed January, 2021 and revised July, 2023).

**Table 1:** *SNHG3 and LUNAR1* genes characteristics retrieved from various bioinformatics databases.

Gene symbol	*SNHG3*	*LUNAR1*
Genomic regions	Chromosome 1—NC_000001.11	Chromosome 15—NC_000015.10
	
HGNC	https://www.genenames.org/data/gene-symbol-report/#!/hgnc_id/HGNC:10118	https://www.genenames.org/data/gene-symbol-report/#!/hgnc_id/HGNC:51199
Chromosomal location	**1p35.3 sense**	**15q26.3 + **
Genomic location by genecards		
Ensembl gene	ENSG00000242125	ENSG00000278090
NCBI Gene ID	8420	104,564,224
Gene product	LncRNA SNHG3	LncRNA LUNAR1
Transcript genome Data Viewer-	https://www.ncbi.nlm.nih.gov/genome/gdv/browser/gene/?id=8420	https://www.ncbi.nlm.nih.gov/genome/gdv/browser/gene/?id=104564224

HGNC; HUGO Gene Nomenclature Committee https://www.genenames.org/ NCBI; National Center for Biotechnology Information https://www.ncbi.nlm.nih.gov/ Ensembl Databases https://www.ensembl.org/index.html (Accessed January, 2021 and revised July, 2023).

### Participants data (Table 2)

**Table 2:** Study participants’ groups (control and CRC) demographic, clinical and pathology data.

Group (n)/Characteristics (Unit)	Control^26^	CRC patients (70)	Statistics, *P-value*
Age (Years)	55.96 ± 10.1	54.89 ± 13.54	
≤ 50 (n, %)	7, 26.92%	25, 35.71%	χ2 = 0.6593, NS
> 50 (n, %)	19, 73.08%	45, 64.29%	
Gender			
Male (n, %)	9, 34.62%	30, 42.86%	χ2 = 0.5339, NS
Female (n, %)	17, 65.38%	40, 57.14%	
BMI (Kg/m^2^)	22.8 (21.5–25.03)	23.7 (22.4–25.03)	U = 357, NS NS
Hgb (gm/dL)			
≤ 11 (n, %)	0,0%	32, 45.71%	χ2 = 17.83, *P*1 < 0.0001
> 11 (n, %)	26,100%	38, 54.29%	
Platelet count (x10^3^cell/µL)			
< 150 (n, %)	0,0%	2, 2.86%	χ2 = 5.585, NS
150–450 (n, %)	26,100%	57, 81.42%	
> 450 (n, %)	0,0%	11, 15.72%	
Lymphocytes (x10^3^cell/µL)			
≤ 5 (n, %)	26, 100%	70, 100%	NA
> 5 (n, %)	0, 0%	0, 0%	
PT (sec)			
≤ 14 (n, %)	26, 100%	58, 82.86%%	χ2 = 5.094, *P1* = *0.024*
> 14 (n, %)	0, 0%	12, 17.14%%	
PLR ratio	106 (91.96- 122)	134 (104.4- 172)	U = 571, *P*2 = 0.0048
LFTs			
ALT (IU/l)	18.5 (13.8- 26.25)	17 (12- 24)	U = 816, NS NS
AST (IU/l)	19.5 (13.5- 24.25)	18.50 (14- 27)	U = 866.5, NS NS
Kidney function tests			
Serum Urea (mg/dL)	27.5 (20.5–31.25)	30 (21–36.25)	U = 746, NS
Serum Creatinine (mg/dL)	0.8527 ± 0.185	0.7709 ± 0.3248	T = 1.211, NS NS
Non-Communicable Diseases			
DM (n, %)			
Yes (n, %)	0,0%	22, 31.43%	χ2 = 10.60, *P1* = 0.0011
No (n, %)	26,100%	48, 68.57%	
HTN (n, %)			
Yes (n, %)	0,0%	7, 10.00%	χ2 = 2.804, NS
No (n, %)	26,100%	63, 90.00%	
CRC Family History			
Yes (n, %)	0,0%	13, 18.57%	χ2 = 5.585, *P1* = 0.0181
No (n, %)	26, 100%	57, 81.43%	
Tumor site			
Colon (n, %)	-	45, 64.29%	NA
Rectum (n, %)	-	19, 27.14%	
Rectosigmoid (n, %)	-	6, 8.57%	
Tumor size (cm)			
≤ 5 (n, %)	-	36, 51.43%	NA
> 5 (n, %)	-	34, 48.57%	
Mucinous or not			
Yes (n, %)	-	16, 22.86%	NA
No (n, %)	-	54, 77.14%	
Tumor invasion			
T1, T2 (n, %)	-	22, 31.43%	NA
T3, T4 (n, %)	-	48, 68.57%	
LNM			
Yes (n, %)	-	23, 32.86%	NA
No (n, %)	-	47, 67.14%	
Vascular invasion			
Yes (n, %)	-	23, 32.86%	NA
No (n, %)	-	47, 67.14%	
Tumor differentiation			
Well differentiated (n, %)	-	16, 22.86%	NA
Moderately differentiated (n, %)	-	42, 60%	
Poorly differentiated (n, %)	-	12, 17.14%	
Signet ring cell feature presence			
Yes (n, %)	-	6, 8.57%	NA
No (n, %)	-	64, 91.43%	
TNM stage			
I-II (early stage) (n, %)	-	47, 67.14%	NA
III-IV (late stage) (n, %)	-	23,32.86%	
Ulcerative colitis			
Yes (n, %)	-	34, 48.57%	NA
No (n, %)	-	36, 51.43%	

Data are mean ± SD (parametric data), median (IQR) (25th percentile-75th percentile) (non-parametric data) or (n, %). Using GraphPad prism software, statistics were computed using Chi square test χ2, Student T test (parametric data) or Mann Whitney U test (non-parametric data), P1 comparison between control and CRC groups n, using Chi square test. While P2, comparison between the mean of the population in control and CRC groups using Student T test or comparison between the median of the population in control and CRC groups using Mann Whitney U test. Statistical significance is at P-value < 0.05. [N.A, not applicable; NS, non-significant; ALT, Alanine aminotransferase; AST, Aspartate aminotransferase; BMI, Body mass index; CA19-9, Carbohydrate antigen 19–9; CEA, Carcinoembryonic antigen; CRC, Colorectal cancer; DM, Diabetes mellitus; Hgb, Hemoglobin; HTN, Hypertension; PT, Prothrombin time; PLR, Platelet-to-lymphocyte ratio; LFTs, Liver function tests; LNM, lymph node metastasis; TNM, tumor-node-metastasis.]

#### Participants demographic and clinical data

Table 2 showed the difference in demographic and clinical data between the CRC patients’ group and the control group, where no statistical significance was detected regarding age, gender, and BMI between the two groups (case-controlled study). However, the CRC patients significantly had low Hgb levels (≤ 11 gm/dl) when compared to the control group (P < 0.0001). Additionally, PLR was also significantly elevated in CRC group only (P = 0.0048). Prolonged PT (> 14 s) was significantly detected in CRC group (P = 0.024).

#### CRC patients group pathological data

According to colonoscopy and radiographic findings, the most common CRC sites were respectively the colon in 45 (64.29%), rectum in 19 (27.14%) and rectosigmoid in 6 (8.57%) of patients. The enrolled CRC patients were divided into two stages where 47 (67.14%) of patients were in early stage (I–II). While 23 (32.86%) patients were in late stage (III–IV). The most common tumor differentiation was moderate differentiation, which was reported in 42 (60%) of the CRC individuals.

### Expression pattern of the investigated lncRNAs in CRC Egyptian patients’ cohort (n = 70) (Fig. 1)

**Fig. 1:**
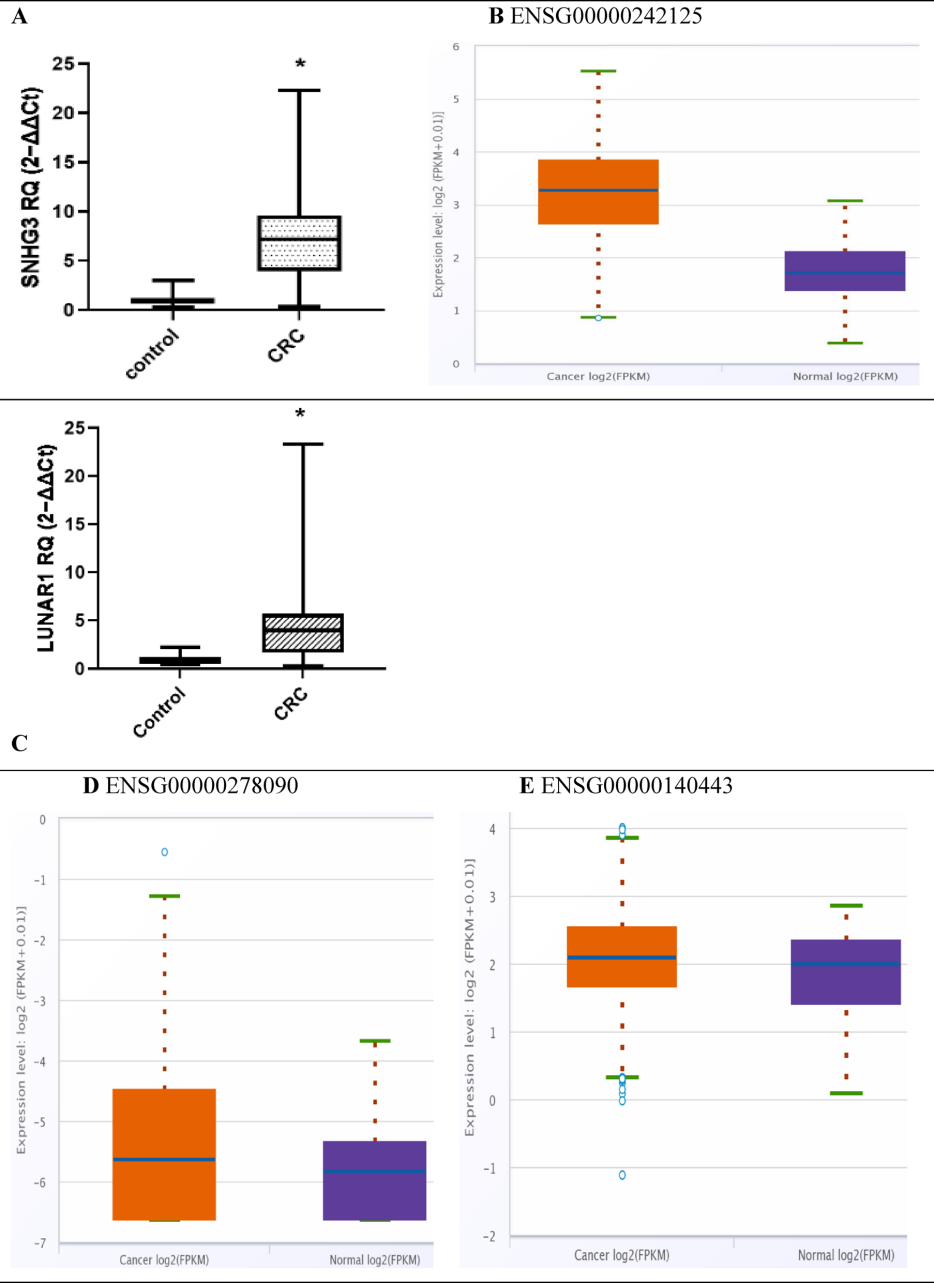
The expression pattern of tested lncRNAs fold-change presented as clustered bar chart (**A**) SNHG3 in CRC patients compared to control, (**B**) SNHG3 lncRNA differential gene expression from ENCORI project in colon adenocarcinoma primary tumors (n = 471) vs control samples (n = 41) https://rnasysu.com/encori/panGeneDiffExp.php, (**C**) LUNAR1 lncRNA fold-change in CRC patients compared to control, (**C** and **D**) Pan-Cancer Differential Expression analysis https://rnasysu.com/encori/panGeneDiffExp.php from ENCORI project samples in colon adenocarcinoma primary tumors (n = 471) vs control samples (n = 41) box blot for LUNAR1 lncRNA (**D**) and IGF1R (**E**). All data were plotted graphically using GraphPad Prism software. Data shown as Box Plot median (IQR) (25th percentile-75th percentile). * Significantly different at *P* < 0.05.

As demonstrated in Fig. 1A. The qRT-PCR data revealed that the serum expression levels of SNHG3 lncRNA fold-change in CRC patients were 7.187(3.940- 9.596) and 1.035(0.6406- 1.149) in controls which was significantly greater in CRC patients’ group (P < 0.0001). This is in line with the in silico database search result via Pan-Cancer Differential Expression Analysis of Genes across ENCORI project Colon adenocarcinoma samples box blot findings (Fig. 1B) https://rnasysu.com/encori/panGeneDiffExp.php (Revised access on June 2023) that was higher in the tumor cases group in comparison to the control group.

As presented in Fig. 1C, likewise, lncRNA LUNAR1 serum expression level was also markedly increased in CRC patients with median of 3.945 (1.668- 5.677) than that in healthy participants with median of 0.7785 (0.500- 1.206) (P < 0.0001).

Pan-Cancer Differential Expression Analysis (Accessed and revised on June 2023) https://rnasysu.com/encori/panGeneDiffExp.php from ENCORI project in colon adenocarcinoma primary tumors (n = 471) vs control samples (n = 41) (Fig. 1D) LUNAR1 lncRNA and (Fig. 1E) IGF1R differential gene expression.

### Demographic and clinical data of CRC patients’ cohort (n = 70) sub-classified according to CRC stages (Table 3)

**Table 3:** CRC patients demographics and clinical data sub-classified as CRC early-stages (I–II) (n = 47) vs CRC late stages (III–IV) (n = 23).

Characteristics	CRC stage		Statistics,
(Unit)/ group (n)	Early (I-II)^47^	Late- (III-IV)^23^	*P-value*
Age (Years)			
≤ 50 (n, %)	18, 38.30%	7, 30.43%	χ2 = 0.4159, NS
> 50 (n, %)	29, 61.70%	16, 69.57%	
Gender			
Male (n, %)	19, 40.43%	11, 47.83%	χ2 = 0.3454, NS
Female (n, %)	28, 59.57%	12, 52.17%	
BMI (Kg/m2)	23.15 (22.4–24.45)	24.29 (22.6–27.28)	U = 125, NS
Hgb (gm/dL)	11.17 (11.2 ± 1.28)	10.64 (10.64 ± 2.6)	T = 1.158, NS
Platelet count (x10^3^cell/µL)	302.0 (220- 377)	371.0 (299- 450)	U = 365.5, 0.0281 *
Lymphocytes (x10^3^cell/µL)	2.647 (2.65 ± 0.914)	2.240 (2.24 ± 0.89)	T = 1.766, NS
PLR ratio	116.8 (94.1- 161.6)	152.0 (133.5- 186.3)	U = 308, 0.0032
LFTs			
ALT (IU/l)	19.94 (19.94 ± 9.6)	18.22 (18.2 ± 12.35)	T = 0.6369, NS
AST (IU/l)	20.00 (13- 28)	18.00 (15- 22)	U = 531, NS
Kidney function tests			
Serum Urea (mg/dL)	30.00 (21- 38)	31.00 (26- 35.3)	U = 447, NS
Serum Creatinine (mg/dL)	0.7291(0.73 ± 0.35)	0.8561 (0.86 ± 0.25)	T = 1.551, NS
Non-communicable diseases			
DM (n, %)			
Yes (n, %)	15, 31.91%	7, 30.43%	χ2 = 0.01570, NS
No (n, %)	32, 68.09%	16, 69.57%	
HTN (n, %)			
Yes (n, %)	6, 12.77%	1, 4.35%	χ2 = 0.82, NS
No (n, %)	41, 87.23%	22, 95.65%	
CRC Family History			
Yes (n, %)	10, 21.28%	3, 13.04%	χ2 = 0.6922, NS
No (n, %)	37, 78.72%	20, 86.96%	
Tumor site			
Colon (n, %)	31, 65.96%	14, 60.87%	χ2 = 3.540, NS
Rectum (n, %)	14, 29.79%	5, 21.74%	
Rectosigmoid (n, %)	2, 4.25%	4, 17.39%	
Tumor size (cm)			
≤ 5 (n, %)	27, 57.45%	9, 39.13%	χ2 = 2.074, NS
> 5 (n, %)	20, 42.55%	14, 60.87%	
Mucinous or not			
Yes (n, %)	10, 21.28%	6, 26.09%	χ2 = 0.2027, NS
No (n, %)	37, 78.72%	17, 73.91%	
Tumor invasion			
T1, T2 (n, %)	22, 46.81%	0, 0%	χ2 = 15.70, < 0.0001
T3, T4 (n, %)	25, 53.19%	23, 100%	
Vascular invasion			
Yes (n, %)	8, 17.02%	15, 65.22%	χ2 = 16.26, < 0.0001
No (n, %)	39, 82.98%	8, 34.78%	
Tumor differentiation			
Well differentiated (n, %)	11, 23.40%	5, 21.74%	χ2 = 1.967, NS
Moderately differentiated (n, %)	26, 55.32%	16, 69.57%	
Poorly differentiated (n, %)	10, 21.28%	2, 8.69%	
Signet ring cell feature presence			
Yes (n, %)	3, 6.38%	3, 13.04%	χ2 = 0.8742, NS
No (n, %)	44, 93.62%	20, 86.96%	
Ulcerative colitis			
Yes (n, %)	21, 44.68%	13, 56.52%	χ2 = 0.8668, NS
No (n, %)	26, 55.32%	10, 43.48%	
CEA (ng/ml)	3.700 (2.8- 6.1)	10.00 (4.5- 26.2)	U = 279, 0.0008
CA19-9 (U/ml)	8.500 (3.2- 13.8)	18.40 (3.9- 101.7)	U = 370.5, 0.0330
LncRNA SNHG3 fold-change (Fold Change)	6.498 (3.18- 8.815)	8.938 (5.69- 11.8)	U = 351.5, 0.0175
LncRNA LUNAR1 fold-change (Fold Change)	3.891 (1.39–5.35)	4.627 (2.828–8.05)	U = 430, NS

Data are mean ± SD (parametric data), median (IQR) (25th percentile-75th percentile) (non-parametric data) or (n, %). Using GraphPad prism software, statistics were computed using Chi square test χ2, Student T test (parametric data) or Mann Whitney U test (non-parametric data). Statistical significance is at *P-*value < 0.05. [N.A, not applicable; NS, non-significant; ALT, Alanine aminotransferase; AST, Aspartate aminotransferase; CA19-9, Carbohydrate antigen 19–9; CEA, Carcinoembryonic antigen; CRC, Colorectal cancer; DM, Diabetes mellitus; Hgb, Hemoglobin; HTN, Hypertension; PLR, Platelet-to-lymphocyte ratio; LFTs, Liver function tests; LNM, lymph node metastasis.]

Late-stage CRC (III-IV) was noticeably associated with higher levels of platelet count with median (IQR) (25th percentile to 75th percentile) of 371.0 (299.0–450.0) than those of early stage CRC (I–II) 302.0 (220.0–377.0) (P = 0.0281). The PLR was markedly elevated in late-stage CRC (III–IV) individuals with median of 152.0 (133.5–186.3) in contrast to early-stage CRCs (I–II) 116.8 (94.08–161.6) (P = 0.0032). The presence of tumor vascular invasion, which was identified in 15 (65.22%) of the late-stage CRC patients group compared to only 8 (17.02%) of the early stage patients, was statistically related to late stage CRC (III–IV) group (P < 0.0001). Additionally, late-stage CRC (III–IV) was significantly connected to a deeper tumor invasion T3, T4 (P < 0.0001). CEA and CA19.9 serum levels were elevated in late-stage CRC (III–IV) group than early-stage ones (I-II), showing statistically difference between the two groups with P = 0.0008 and P = 0.033, respectively.

The expression level of lncRNA SNHG3 was substantially increased in late-stage CRC patients (III-IV) with median of 8.938 (5.696–11.79) than those of early-stage CRC (I–II) with median of 6.498 (3.182–8.815) (P = 0.0175), demonstrating statistically distinction between the two stages. Finally, the expression level of LUNAR1 didn’t reveal statistically difference between the early and late-stage CRC groups (P > 0.05).

### Expression pattern of lncRNA fold-change in relation to patients’ clinico-pathological characteristics cut-off (Table 4)

**Table 4:** The expression pattern of SNHG3 and LUNAR1 lncRNAs fold-change in relation to CRC patients’ (n = 70) according to demographic and clinico-pathological charachteristics cut-offs.

Characteristics cut-off	lncRNA fold-change			
(Unit)	SNHG3	Statistics, adjusted *p*	LUNAR1 **A**	Statistics, adjusted *p*
Age (Years)				
≤ 50	6.916 (4.200–9.782)	U = 560, NS	3.732 (1.728–5.118)	U = 534, NS
> 50	7.413 (3.482–9.320)		4.00 (1.564–6.635)	
Gender				
Male	7.837 (5.531–9.530)	U = 533, NS	3.031 (1.068–5.353)	U = 455.5, NS
Female	6.175 (3.387–9.850)		4.362(2.681–6.658)	
Hgb (gm/dL)				
≤ 11	7.163 (4.005–9.850)	U = 576, NS	3.706 (1.267–5.446)	U = 512, NS
> 11	7.343 (3.904–9.223)		4.214 (2.530–8.014)	
Platelet count (x103cell/µL)				
< 150	6.261 (3.074–9.448)	H = 1.093, NS	3.206 (1.753–4.659)	H = 0.8568, NS
150–450	6.964 (4.200–9.580)		3.732 (1.404–5.696)	
> 450	8.815 (3.506–11.79)		4.823 (3.031–6.681)	
Non-communicable diseases				
DM				
Yes	7.163 (4.079–9.547)	U = 527, NS	3.866 (2.323–5.618)	U = 508.5, NS
No	7.213 (3.651–9.833)		4.016 (1.499–6.057)	
HTN				
Yes	9.448 (2.071–11.79)	U = 182.5, NS	3.482 (2.657–4.659)	U = 204.5, NS
No	6.964 (4.084–9.514)		4.00 (1.414–6.190)	
CRC family history				
Yes	5.242 (2.848- 9.164)	U = 262, NS	3.482 (0.8179–6.479)	U = 306, NS
No	7.674 (5.129- 9.782)		4.00 (2.123–5.696)	
Tumor site				
Colon	6.453 (3.482–8.877)	H = 2.605, NS	3.891 (1.404–5.696)	H = 0.07334, NS
Rectum	8.754 (5.242–10.48)		4.00 (2.639–6.19)	
Rectosigmoid	9.032 (5.962–12.44)		4.252 (1.749–6.376)	
Tumor size (cm in length)				
≤ 5	5.242 (2.888–8.282)	U = 254, *P* < 0.0001	2.868 (1.134–4.637)	U = 365, *P* = 0.0033
> 5	8.938 (6.964–11.39)		4.774 (3.370 -6.681)	
Mucinous or not				
Yes	9.032 (4.068–10.43)	U = 355.5, NS	4.275 (2.700–5.366)	U = 416.5, NS
No	6.940 (3.940–9.223)		3.866 (1.390–6.313)	
Tumor invasion				
T1, T2	5.521 (3.109–7.710)	U = 307, *P* = 0.0046	3.140 (1.063–4.675)	U = 367.5, *P* = 0.0420
T3, T4	8.518 (5.512–11.24)		4.317 (2.196–6.681)	
LNM				
Yes	8.938 (5.696–11.79)	U = 351.5, *P* = 0.0175	4.627 (2.828–8.056)	U = 430, NS
No	6.498 (3.182–8.815)		3.891 (1.395–5.352)	
Vascular invasion				
Yes	8.938 (6.063–12.91)	U = 290.5, *P* = 0.0015	4.627 (2.828- 6.681)	U = 416, NS
No	6.453 (3.182–8.938)		3.482 (1.395- 5.278)	
Tumor differentiation				
Well differentiated	7.647 (4.142–9.63)	H = 1.676, NS	3.366 (1.557- 4.106)	H = 3.534, NS
Moderately differentiated	7.543 (4.808–10.56)		4.724 (2.530- 6.612)	
Poorly differentiated	5.719 (2.235–9.079)		3.294 (0.6607- 4.355)	
Signet ring cell feature presence				
Yes	9.353 (4.858- 12.18)	U = 139, NS	4.153 (0.9941–9.535)	U = 191.5, NS
No	6.989 (3.651- 9.497)		3.945 (1.825–5.716)	
Ulcerative colitis				
Yes	7.543 (5.316–9.207)	U = 581.5, NS	4.643 (2.544–6.681)	U = 465.5, NS
No	6.989 (3.382–10.34)		3.366 (1.085–4.925)	

Data are median (IQR) (25th percentile-75th percentile). Using GraphPad prism software, statistics were computed using Mann Whitney U test (non-parametric data) to determine significant variation between two groups, Kruskal–Wallis (H) (non-parametric data) to determine significant variations between multiple groups, followed by Dunn’s multiple comparison test, when required. Statistical significance is at *P-*value < 0.05. [NS, Non-significant.]

#### SNHG3 lncRNA (Fig. 2)

**Fig. 2:**
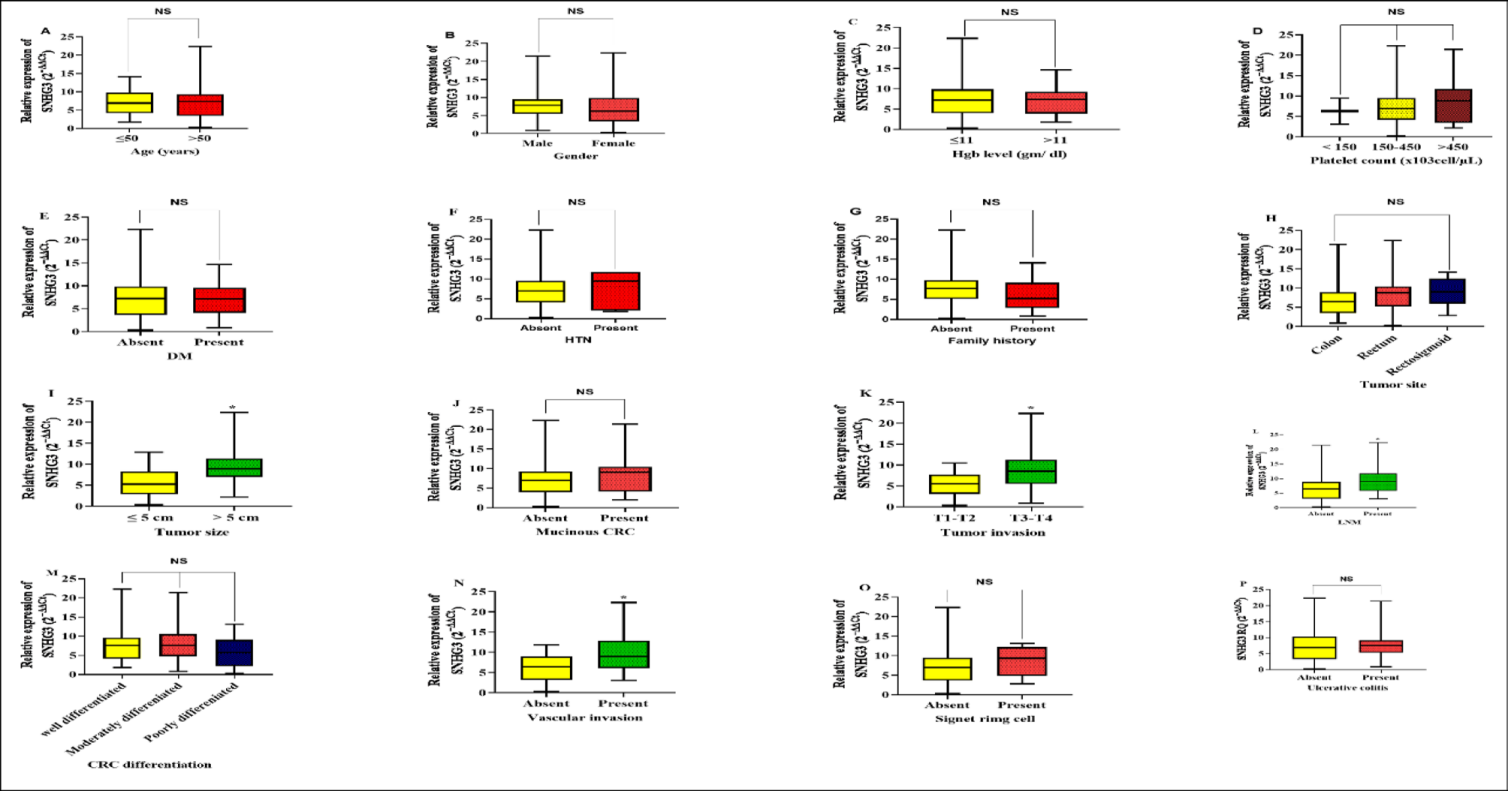
The relative expression pattern of lncRNA SNHG3 in relation to CRC patients’ demographics and clinicopathological characteristics; (**A**) Relative expression of lncRNA SNHG3 in CRC patients with age ≤ 50 years and those with age > 50 years. (**B**) Relative expression of lncRNA SNHG3 in male CRC patients compared to female CRC patients. (**C**) Relative expression of lncRNA SNHG3 in CRC patients with Hgb level ≤ 11 gm/dl and those with Hgb level > 11 gm/dl. (**D**) Relative expression of lncRNA SNHG3 among various platelet counts in CRC patients. (**E**) Relative expression of lncRNA SNHG3 in CRC patients who exhibiting history of DM or not. (**F**) Relative expression of lncRNA SNHG3 in CRC patients who exhibiting history of HTN or not. (**G**) Relative expression of lncRNA SNHG3 in CRC patients who had family history of CRC or not. (**H**) Relative expression of lncRNA SNHG3 among the various tumor sites of CRC tumor. (**I**) Relative expression of lncRNA SNHG3 in CRC patients with tumor size ≤ 5 cm and those with tumor size > 5 cm in length. (**J**) Relative expression of lncRNA SNHG3 in CRC patients who exhibiting mucinous tumor or not. (**K**) Relative expression of lncRNA SNHG3 in CRC patients with T1–T2 tumor invasion depth and those with deeper invasion T3–T4. (**L**) Relative expression of lncRNA SNHG3 in CRC patients who exhibiting LNM or not. (**M**) Relative expression of lncRNA SNHG3 maong the various differentiation status of CRC tumors. (**N**) Relative expression of lncRNA SNHG3 in CRC patients who exhibiting vascular invasion or not. (**O**) Relative expression of lncRNA SNHG3 in CRC patients who exhibiting signet ring cell feature or not. (**P**) Relative expression of lncRNA SNHG3 in CRC patients who exhibiting history of ylcerative colitis or not. All data was plotted graphically using GraphPad Prism software. Data is shown as Box Plot median (IQR) (25th percentile-75th percentile). Statistics were computed using Mann–Whitney test (non-parametric data). *Significantly different is at *P* < 0.05.

An increased expression level of SNHG3 was significantly related to the greater tumor size > 5 cm with median of 8.938 (6.964–11.39) compared to 5.242 (2.888–8.282) in the smaller tumor size ≤ 5 cm (P < 0.0001). Additionally, the greater extensive invasion depth T3, T4 was substantially associated with higher expression of SNHG3 with median 8.518 (5.512–11.24) in comparison to 5.521 (3.109–7.710) in T1, T2 tumor invasion depth (P = 0.0046). Moreover, SNHG3 high expression was revealed to be strongly linked to the presence of tumor vascular invasion with median fold change of 8.938 (6.063–12.91) in contrast to 6.453 (3.182–8.938) in negative tumor vascular invasion CRCs (P = 0.0015). Moreover, patients who had LNM exhibited a significantly higher expression level of SNHG3 with median 8.938 (5.696–11.79) than those who did not with median of 6.498 (3.182–8.815) (P = 0.0175).

#### LUNAR1 lncRNA (Fig. 3)

**Fig. 3:**
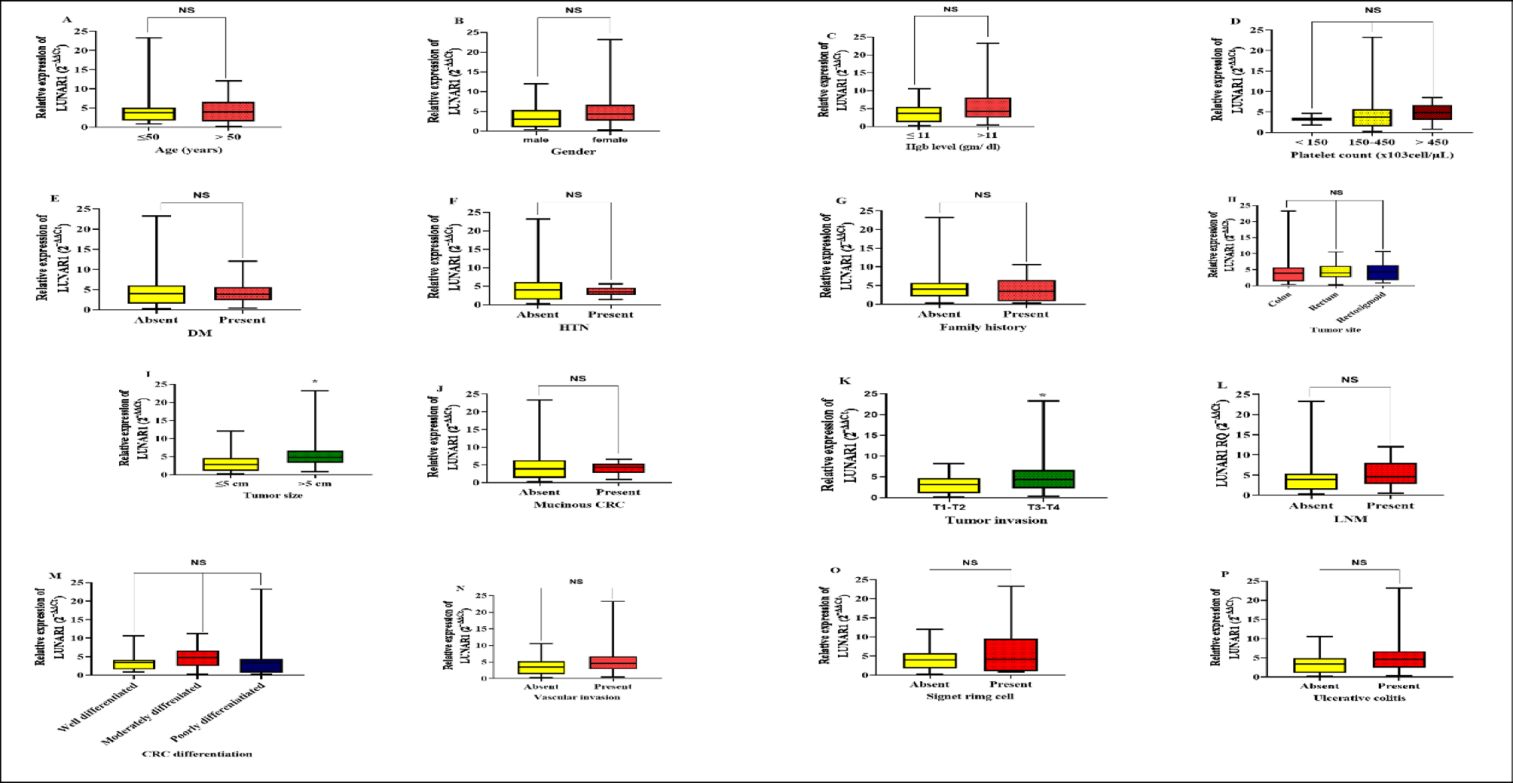
The relative expression pattern of lncRNA LUNAR1 in relation to CRC patients’ demographics and clinicopathological charachteristics; (**A**) Relative expression of lncRNA LUNAR1 in CRC patients with age ≤ 50 years and those with age > 50 years. (**B**) Relative expression of lncRNA LUNAR1 in male CRC patients compared to female CRC patients. (**C**) Relative expression of lncRNA LUNAR1 in CRC patients with Hgb level ≤ 11 gm/dl and those with Hgb level > 11 gm/dl. (**D**) Relative expression of lncRNA LUNAR1 among various platelet counts in CRC patients. (**E**) Relative expression of lncRNA LUNAR1 in CRC patients who exhibiting history of DM or not. (**F**) Relative expression of lncRNA LUNAR1 in CRC patients who exhibiting history of HTN or not. (**G**) Relative expression of lncRNA LUNAR1 in CRC patients who had family history of CRC or not. (**H**) Relative expression of lncRNA LUNAR1 among the various tumor sites of CRC tumor. (**I**) Relative expression of lncRNA LUNAR1 in CRC patients with tumor size ≤ 5 cm and those with tumor size > 5 cm in length. (**J**) Relative expression of lncRNA LUNAR1 in CRC patients who exhibiting mucinous tumor or not. (**K**) Relative expression of lncRNA LUNAR1 in CRC patients with T1-T2 tumor invasion depth and those with deeper invasion T3-T4. (**L**) Relative expression of lncRNA LUNAR1 in CRC patients who exhibiting LNM or not. (**M**) Relative expression of lncRNA LUNAR1 maong the various differentiation status of CRC tumors. (**N**) Relative expression of lncRNA LUNAR1 in CRC patients who exhibiting vascular invasion or not. (**O**) Relative expression of lncRNA LUNAR1 in CRC patients who exhibiting signet ring cell feature or not. (**P**) Relative expression of lncRNA LUNAR1 in CRC patients who exhibiting history of ulcerative colitis or not. All data was plotted graphically using GraphPad Prism software. Data is shown as Box Plot median (IQR) (25th percentile-75th percentile). Statistics were computed using Mann–Whitney test (non-parametric data). *Significantly different is at P < 0.05.

CRC patients who had larger tumor size > 5 cm displayed a significantly higher expression level of LUNAR1 with median (IQR) (25th percentile-75th percentile) 4.774 (3.370–6.681) than those with smaller tumor size ≤ 5 cm with median of 2.868 (1.134–4.637) (P = 0.0033). Additionally, greater expression of LUNAR1 was substantially correlated to extensive tumor invasion depth T3, T4 with median of 4.317(2.196–6.681) compared to 3.140 (1.063–4.675) in T1, T2 invasion depth (P = 0.0420).

### LncRNAs SNHG3, LUNAR1, CEA and CA19.9 correlation with CRC patients (n = 70) clinico-pathological characteristics (Table 5)

**Table 5:** Spearman correlation coefficient between CRC patients (n = 70) clinico-pathological features and lncRNAs SNHG3 and LUNAR1 expression levels.

Characteristics	Correlation coefficient	LncRNAs		Prognostic TMs	
(Unit)		SNHG3	LUNAR1	CEA	CA19.9
Platelets	Spearman	0.1801	0.07365	0.2841	0.1055
(x10^3^cell/µL)	*p-value*	NS	NS	0.0172	NS
PLR	Spearman	0.1378	0.1590	0.2502	0.1592
	*p-value*	NS	NS	0.0367	NS
ALT (IU/L)	Spearman	− 0.05319	− 0.2144	− 0.3232	− 0.0044
	*p-value*	NS	NS	0.0064	NS
Tumor size (Cm)	Spearman	0.5469	0.2672	0.3908	0.05661
	*p-value*	0.0001	0.0253	0.0008	NS
LNM	Point-Biserial	0.260	0.154	0.282	0.320
	*p-value*	0.03	NS	0.018	0.007
Vascular invasion	Point-Biserial	0.416	0.228	0.238	0.278
	*p-value*	0.0001	NS	0.047	0.02

Using graphpad prism software, Spearman correlation coefficient *r* was used to measure the degree of association between two continuous non-parametric variables, however, point-biserial correlation was used to measure the association between two variables; one continuous and the other is dichotomous using SPSS software. Hb, lymphocytes, urea, creatinine, AST, DM, tumor site if colonic or rectal, mucinous type, CRC family history, ulcerative colitis, HTN were all NS. Statistical significance is at *p-*value < 0.05, NS non-significant. [LNM, Lymph Node Metastasis; ALT, Alanine aminotransferase; PLR, Platelet-to-lymphocyte ratio; NS, non-significant.]

As displayed in Table 5, SNHG3 was significantly positively correlated with tumor size (*r* = 0.5469) (*P* < 0.0001), LNM (point-biserial correlation coefficient *r*_*pb*_ = 0.26) (*P* = 0.03) and vascular invasion (*r*_*pb*_ = 0.416) (*P* < 0.0001). On the other hand, LUNAR1 expression was found to be positively correlated with tumor size (*r* = 0.2672) (*P* = 0.0253). The classical TM, CEA was positively correlated with platelet counts of CRC patients (*r* = 0.2841) (*P* = 0.0172), PLR (*r* = 0.2502) (*P* = 0.0367), tumor size (*r* = 0.3908) (*P* = 0.0008), lymph node metastases (*r*_*pb*_ = 0.282) (*P* = 0.018) and presence of vascular invasion (*r*_*pb*_ = 0.238) (*P* = 0.047). In contrast, CEA showed a negative correlation with ALT (IU/L) (*r* = − 0.3232) (*P* = 0.0064). While CA19.9 was only showed a positive correlation with lymph node metastases (*r*_*pb*_ = 0.320) (*P* = 0.007) and presence of vascular invasion (*r*_*pb*_ = 0.278) (*P* = 0.02). Other demographic and clinicopathological characteristics were non-significant (Supplementary Table S1) (age, gender, hemoglobin, lymphocytes, AST, urea, creatinine, DM, tumor site, HTN, CRC family history, and ulcerative colities).

### The correlation coefficient between lncRNAs SNHG3 and LUNAR1 and the conventional classical CRC TMs (CEA and CA19-9) (Table 6)

**Table 6:** The correlation coefficient between CRC classical prognostic TMs and lncRNAs SNHG3 and LUNAR1 among the CRC patients group (n = 70).

CRC TMs	Correlation	lncRNA fold-change expression	
	coefficient	SNHG3	LUNAR1
CEA (ng/ml)	*95% CI*	(0.1278–0.5522)	(0.1448–0.5641)
	*r, p-value*	0.3584, 0.0023	0.3734, 0.0015
CA19-9 (U/ml)	*95% CI*	(0.006927–0.4621)	(-0.09710 -0.3762)
	*r, p-value*	0.2482, 0.0383	0.1480, NS

The correlation coefficient was determined using the GraphPad prism software and determined by Spearman correlation *r* test, Statistical significance is at *P-*value < 0.05. [NS, non-significant.]

In order to determine the relationship between the fold change expression of lncRNAs SNHG3 and LUNAR1 in the CRC patients’ cohort (n = 70), the Spearman *r* correlation coefficient was used. Additionally, a considerably linear positive correlation was revealed between SNHG3 expression fold change and the classical TMs, CEA (*r* = 0.3584) (*P* = 0.0023) as well as CA19.9 (*r* = 0.2482) (*P* = 0.0383). Moreover, expression fold change of LncRNA LUNAR1 was exhibited to be positively correlated with CEA (*r* = 0.3734) (*P* = 0.0015). On the other hand, LUNAR1 expression fold change did not significantly correlate linearly with CA19.9.

**Fig. 4:**
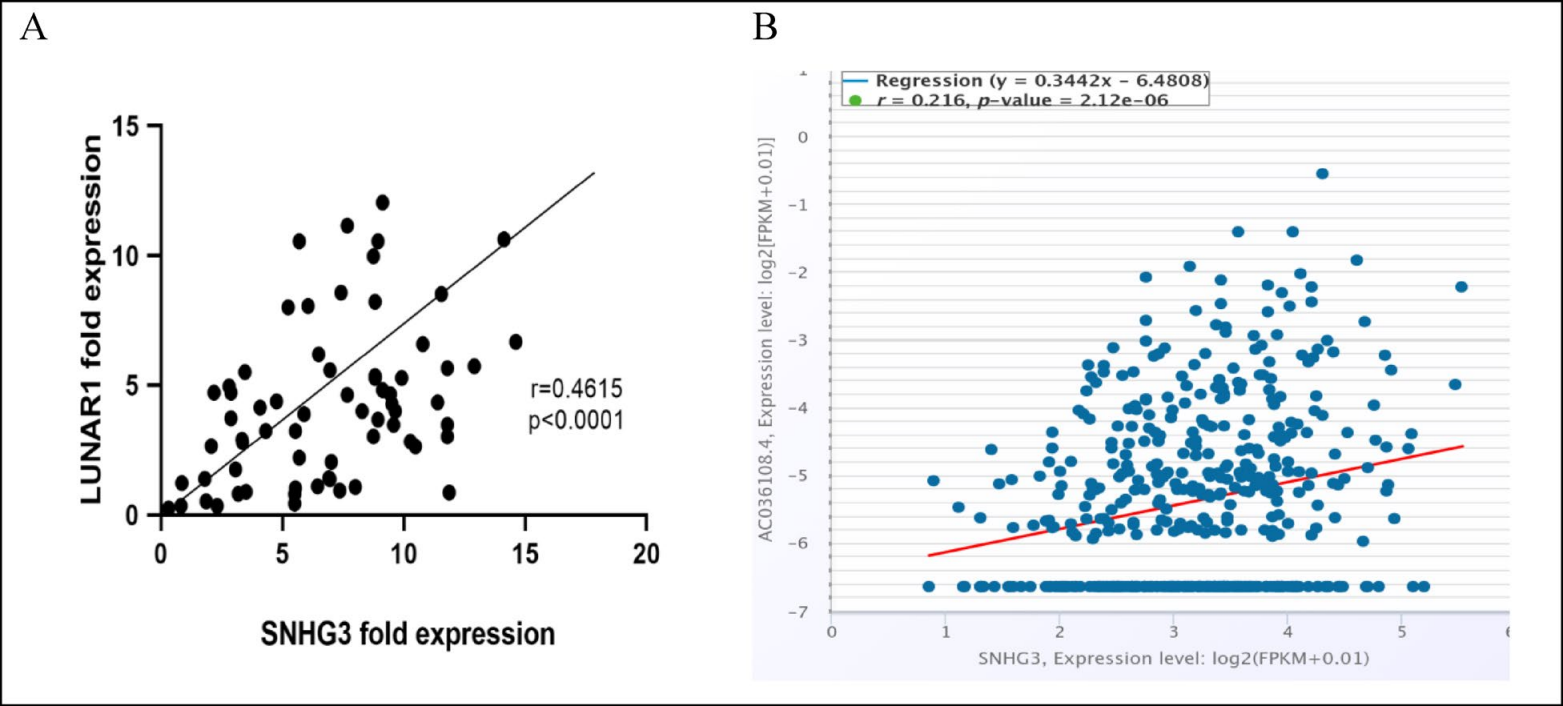
(**A**) LncRNAs SNHG3 and LUNAR1 fold-change expression levels correlations in CRC group (n = 70). At 95% C.I (0.2474–0.6326), Spearman correlation coefficient (r) was calculated using GraphPad Prism software. Statistical significance is at P-value < 0.05. (**B**) Co-Expression Analysis image via bioinformatics for SNHG3 ENSG00000242125 as the query gene and LUNAR1:AC036108.4 ENSG00000278090 as the target gene via the Pan-Cancer Co-Expression Analysis for the RNA-RNA interactions using 471 Colon cancer samples from the ENCORI project. Retrieved from https://rnasysu.com/encori/panGeneCoExp.php.

In Fig. 4A a substantial positive correlation was demonstrated between the fold change expression of lncRNAs SNHG3 and LUNAR1 (*r* = 0.4615) (*P* < 0.0001) which is in line with Fig. 4B in silico Pan-Cancer Co-expression analysis https://rnasysu.com/encori/panGeneCoExp.php, again, in 471 CRC samples ENCORI project.

### Multiple regression analysis for determining predictors of lncRNAs SNHG3 and LUNAR1 increased expression (Table 7)

**Table 7:** Multiple regression analysis for predicting factors affecting lncRNAs SNHG3 and LUNAR1 expression levels in the CRC patients’ cohort group (n = 70) as the dependent variable vs demographic data and the clinico-pathological characteristics as the independent variables.

lncRNA as dependent variable				
SNHG3 expression	LUNAR1 expression			
F (13, 56) = 4.40, *P* < 0.0001*, *R*^*2*^ = 0.505	F (13, 56) = 1.611, *P* = NS, *R*^*2*^ = 0.272			

Independent variables (Unit)	Standardized beta	*P*	Standardized Beta	*P*
Gender (M/F), Age (years)	− 0.048, − 0.021	NS	0.224, − 0.037	NS
CRC Family History	− 0.093	NS	0.117	NS
Tumor size (cm)	0.346	0.004	0.037	NS
Tumor Site (colon, rectal or rectosigmoid)	0.104	NS	− 0.001	NS
Lymph node metastasis	− 0.140	NS	0.074	NS
CRC stage	0.222	NS	0.0001	NS
Vascular invasion	0.352	0.009	− 0.074	NS
Mucinous tumors	− 0.060	NS	− 0.104	NS
Ulcerative colitis	− 0.223	NS	0.284	NS
DM, HTN	0.102, 0.123	NS	− 0.048, − 0.091	NS
LUNAR1 expression	0.279	0.009	–	–

Multiple regression analyses were run using SPSS software. Statistical significance is at *P-*value < 0.05, NS non-significant. [CRC, Colorectal cancer; DM, Diabetes mellitus; HTN, Hypertension; NS, non-significant.]

Multiple regression analysis, showed that, in-line with the design of a case-controlled study, no influence of gender and age on either lncRNA. Tumor size, presence of vascular invasion, and LUNAR1 expression were independent factors for SNHG3 expression with standardized beta coefficient of 0.346 (*P* = 0.004), 0.352 (*P* = 0.009), and 0.279 (*P* = 0.009), respectively. While, the expression of SNHG3 was shown to be the only independent variable for LUNAR1 expression with standardized beta coefficient of 0.410 (*P* = 0.009).

### The discriminative utility of the investigated lncRNAs for CRC patients (Table 8)

**Table 8:** Utility of the investigated bio-molecular markers lncRNA SNHG3 and LUNAR1 and the conventional TMs for better identification of CRC cases cohort (n = 70).

%										asymptomatic
Single marker (Unit)	Cut-off	S.E.M	AUC	Accuracy	Sn	Sp	PPV	NPV	95% CI	*P*
SNHG3 fold-change	> 1.8661	0.0180	0.967	0.89	92.86	96.15	98.5	83.3	(0.909–0.993)	< 0.0001
LUNAR1 fold-change	> 1.338	0.0329	0.891	0.72	80.00	92.31	96.6	63.2	(0.811–0.946)	< 0.0001
CEA (ng/ml)	> 4.6	0.0531	0.703	0.32	47.14	84.62	89.2	37.3	(0.601–0.792)	0.0001
CA19-9 (U/ml)	> 12.7	0.0597	0.503	0.05	40.00	65.38	75.7	28.8	(0.399–0.607)	NS
Combined markers										
SNHG3 + LUNAR1	–	0.0190	0.966	0.9187	95.71	96.15	97.0	84.8	(0.908 − 0.992)	< 0.0001
SNHG3 + CEA	–	0.0182	0.966	0.9	94.29	96.15	98.5	86.2	(0.908–0.992)	< 0.0001
SNHG3 + CA19.9	–	0.0175	0.969	0.89	92.86	96.15	98.5	83.3	(0.911–0.9923)	< 0.0001
LUNAR1 + CEA	–	0.032	0.896	0.7615	80.00	96.15	98.2	64.1	(0.816–0.949)	< 0.0001
LUNAR1 + CA19.9	–	0.0331	0.89	0.72	80.00	92.31	96.6	63.2	(0.810–0.945)	< 0.0001

Using MedCalc software, data were acquired from the ROC curve analysis. Statistical significance is at *P-*value < 0.05, asymptomatic 95% C.I expressed as (lower bound—upper bound). [AUC, area under the curve; NPV, negative predictive value; PPV, positive predictive value; S.E.M; Standard Error of the Mean, Sn, sensitivity; Sp, specificity; NS, non-significant.]

**Fig. 5:**
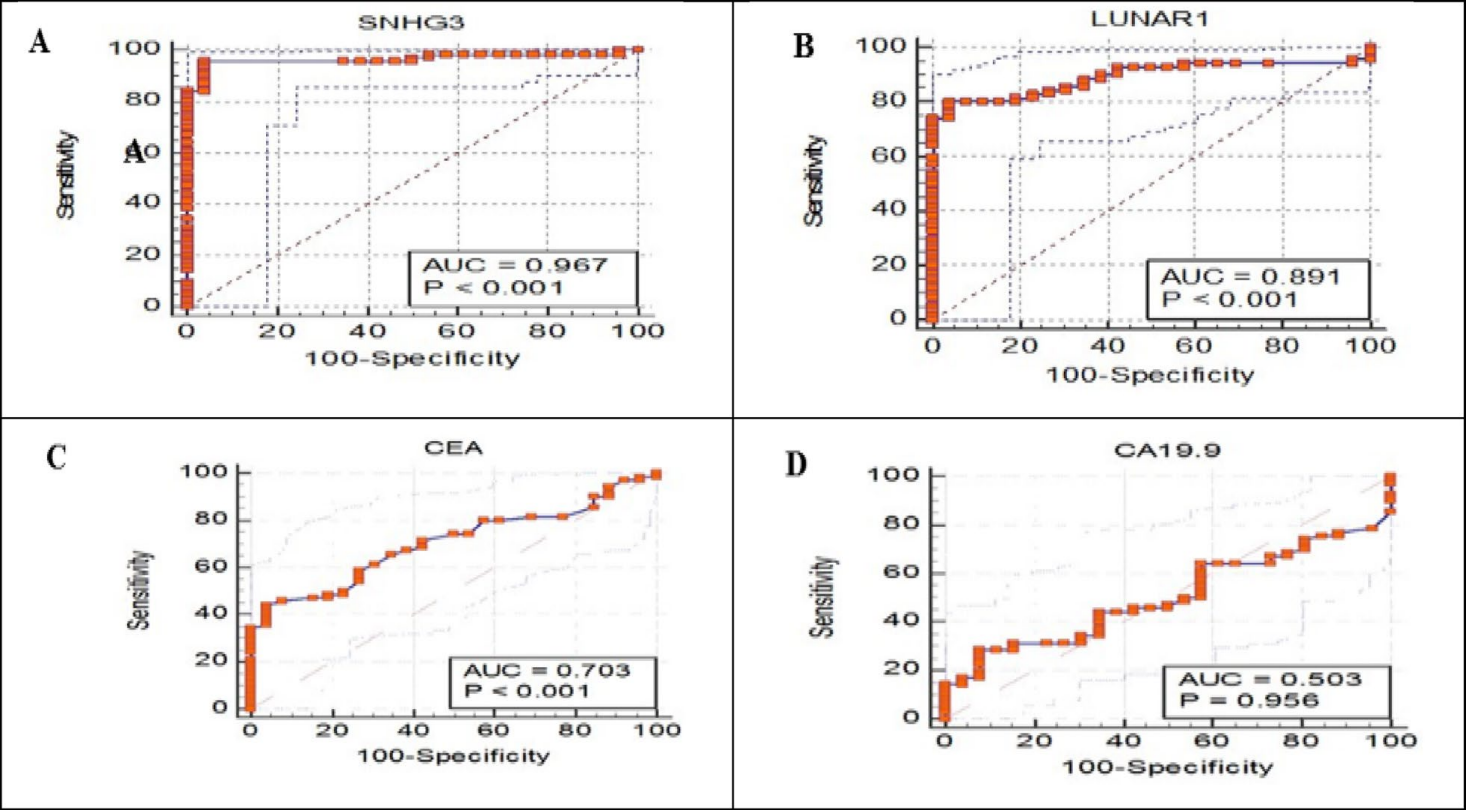
Receiver operating characteristic (ROC) curve for the discriminative utility of lncRNAs SNHG3 (**A**), LUNAR1 (**B**), CEA (**C**), and CA19-9 (**D**) to differentiate CRC cases from the control group. [MedCalc software (https://www.medcalc.org/calc) was used to obtain all values. Data were plotted graphically using MedCalc software. Statistical significance P-value < 0.05.

The potential utility of the studied molecular lncRNAs in comparison to CEA and CA19-9 TMs was assessed, utilizing ROC curve analysis with established cut-off levels for each biomarker. The best cut-off levels which identified CRC patients from controls, was the one that gave SP of 96.15% and SN of 92.86% for lncRNA SNHG3 with AUC of 0.967 (*P* < 0.0001) and gave 80% SN and 92.31% SP for LUNAR1 with AUC of 0.891 (*P* < 0.0001). For the conventional CRC TMs, CEA and CA19.9, SNs were 47.14% and 40%, respectively, however, 84.62% and 65.38%, respectively, SP%, as revealed in Table 8 and Fig. 5A–D for single markers.

The combination of LUNAR1 expression with SNHG3 expression yielded better SN of 95.71% and SP of 96.15% than those for each lncRNA alone. Whenever, the classical TMs CEA and CA19-9 are paired with the lncRNAs as indicators for CRC cases, an improved AUC, SPs, and SNs values were obtained (Table 8). This finding points to the importance of the lncRNAs SNHG3 and LUNAR1 expression measurement for CRC-recurrence monitoring and follow-up after surgery.

The negative LR is illustrated in Table 9, where a value of 0 to 1 denotes the presence of disease. As disorder becomes more probable, negative LR values are more likely to drop, becoming closer to zero, which indicates a greater marker’s SN. This reveals that the lncRNA SNHG3 had the greatest computed SNs and SPs, which were subsequently preceded by the lncRNA LUNAR1, CEA, and then CA19-9. This was established according to the relevance of the AUC out from the ROC curve and the optimal cut-off value obtained for each biomarker, confirming results obtained in Table 8.

**Table 9:** Negative Likelihood Ratios (−LR) of the estimated bio-molecular markers in CRC patients (n = 70).

Markers (Unit)	−LR	*P* value
SNHG3 fold-change	0.074	< 0.0001*
LUNAR1 fold-change	0.22	< 0.0001*
CEA (ng/ml)	0.62	0.0001*
CA19-9 (U/ml)	0.92	NS

Estimated % reduction in probability is large (45%) when − LR = 0.1, moderate (30%) when − LR = 0.1–0.2, and small (15%) when − LR = 0.2–0.5. * Statistical significance is at *P-*value < 0.05, NS non-significant. Distinct significant levels were observed among the analyzed groups when the cut-off values obtained in Table 8, of the evaluated bio-molecular markers were considered. As shown in Table 10, a result was deemed positive or negative for the marker, whether it was above or beneath the cut-off number. In CRC participants exhibiting positive rates in accordance to the cut-off value, the median levels of lncRNA SNHG3, LUNAR1, CEA, and CA19.9 were 7.187-fold change, 3.945-fold change, 4.25 ng/ml and 9.45 U/ml, respectively.

**Table 10:** Cut-off values and median (IQR) (25th percentile-75th percentile) levels with positive rates of the conventional prognostic TMs and the lncRNAs expression levels fold change in the control group (n = 26) and the CRC patients group (n = 70).

Marker (Unit)	Tumor marker				LncRNA fold-change			
	CEA (ng/mL)		CA 19–9 (U/mL)		SNHG3		lncRNA LUNAR1	
	Cut-off	Median	Cut-off	Median	Cut-off	Median	Cut-off	Median
Groups	> 4.6	(IQR)	12.7 >	(IQR)	> 1.8661	(IQR)	> 1.338	(IQR)
Control	4(15.38%)	3.15(2.7–4.25)	9 (34.6%)	10.5(5.57–16.25)	1(3.85%)	0.928(0.64–1.15)	2(7.7%)	0.5607(0.49–1.12)
CRC	33(47.14%)	4.25(3–13.05)	28 (40%)	9.45(3.55–19.45)	65(92.8%)	7.18(3.94–9.6)	56(80%)	3.94(1.668–5.67)

Data are expressed as the positive rates displayed as n (%) and median (25th percentile-75th percentile).

### In silico functional enrichment analysis (accessed august 31st, 2023)

#### Utilizing KEGG

To analyze the functional enrichment of Notch-signaling related genes, diseases, networks, drugs, and pathways, via linkDB search https://www.genome.jp/dbget-bin/get_linkdb?-t+5+ko:K02599 KEGG ORTHOLOGY K02599, 2 drugs from 1 database KEGG DRUG target Notch, and they are: **Crenigacestat** (hydrolase) and **Brontictuzumab** (a monoclonal Antibody).

SNHG3 is targeted by **tamoxifen and sorafenib** according to LncRNA—LncRNAWiki –

CNCB-NGDC https://ngdc.cncb.ac.cn/lncrnawiki/lncrna?symbol=SNHG3

but no drug treatment could be retrieved for LUNAR1 via databases, up till now,

https://ngdc.cncb.ac.cn/lncrnawiki/lncrna?symbol=LUNAR1

#### Gene–gene interactions, gene-protein pathways interaction, and protein–protein interaction (PPI)

**Fig. 6:**
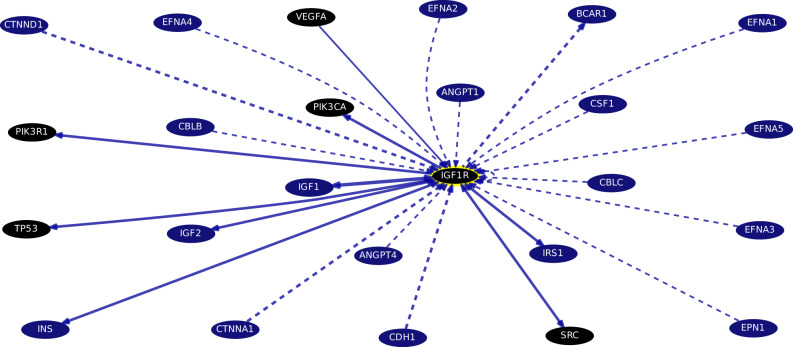
IGF1R most-mentioned/most-curated interactions image retrieved from https://genome.ucsc.edu/cgi-bin/hgGeneGraph?gene=IGF1R&supportLevel=pwy&hideIndirect=on&geneCount=25&geneCount=25&1=OK&geneAnnot=drugbank (Accessed August 31st, 2023). [Interactions colored by support; blue: pathway database, black: annotated genes with drug treatment from DrugBank, dashed lines: interactions without text mining support. VEGFA: vascular endothelial growth factor alpha treated by carvidolol, gliclazide, dalteparin, minocycline, TP53: tumor protein 53 transcription factor affected by acetylsalicylic acid, PIK3R1: phosphoinositide-3-kinase regulatory subunit 1 (alpha) adaptor molecule, kinase modulator, treated by isoprenaline, PIK3CA: phosphatidylinositol-4,5-bisphosphate 3-kinase, catalytic subunit alpha, lipid kinase, treated by caffeine, SRC: v-src-avian sarcoma viral oncogene homolog, non-receptor tyrosine protein kinase, DrugBank Dasatinib, Bosutinib, Ponatinib.]

Figure 6 from curated databases and text-mining showing top genes targets for IGF1R (chr15:99,192,760–99,507,759) interaction with pathway database support retrieved from UCSC Genome project (Accessed on July, 2023).

https://genome.ucsc.edu/cgi-bin/hgGeneGraph?gene=IGF1R&supportLevel=pwy&hideIndirect=on&geneCount=25&geneCount=25&1=OK&geneAnnot=drugbank

## Discussion

Per, Notch-signaling pathway was thought to have a controversial role in CRC evolution, either maintaining healthy intestinal epithelial cells or being involved in the cellular transformation into tumor-promoting cells^51,52^. Therefore, we decided to assess the impact of blood Notch-associated-lncRNAs SNHG3 and LUNAR1 as potential molecular markers involved both in CRC pathogenesis.

Strength-related-to-the-study. To our knowledge, this investigation is the first to examine both SNHG3 and LUNAR1 oncogenes in CRC patients’ peripheral blood, as liquid biopsy, in relation to clinico-pathological tumor features. Moreover, this is the first study showing the correlation between lncRNAs SNHG3 and LUNAR1 blood expression levels as well as their clinical significance as combined model expressed, for CRC follow-up. Results obtained were validated through in silico/bioinformatics analysis.

Studies revealed various cancers overexpress the oncogenic lncRNA SNHG3, where, Zhang L et al. showed that SNHG3 was significantly enhanced in ovarian cancer tissues and cells, accelerating cells’ proliferation and migration through Notch1 stimulation^34^. Liu et al. has pointed out SNHG3 was persistently highly expressed in the serum and tissue samples from ovarian cancer patients being related to LNM and high histological grade^38^. Another study by Ma et al. illustrated an elevated SNHG3 expression in breast cancer tissues linked to tumour aggressiveness of tumor advanced histological grade, TNM stage, LNM, oestrogen receptor, and Her-2 were all associated with SNHG3 lncRNA overexpression^53^. Huang et al. revealed increased SNHG3 expression in CRC tissues, linked to the patients’ poor prognosis. They additionally demonstrated that SNHG3 can function as competitive endogenous (ce)RNA in CRC, boosting CRC progression through regulation of miR-182-5p/Myc axis^35^.

The NOTCH-related lncRNA LUNAR1 exerts a tumorigenic role in cancer as well. Peng & Feng concluded LUNAR1 expression was increased in diffuse large B-cell lymphoma (DLBCL) tissues and cells and its increased level was related to advanced tumor stage and poor patients’ outcome^36^. Nashwa et al. also noted that LUNAR1 and Notch 1 expression levels were up-regulated in pediatric T-cell acute lymphoblastic leukemia being connected to LN infiltration, the presence of minimal residual disease, and poor clinical response with an unfavorable outcome^54^ Additionally, Riahi et al. determined LUNAR1 overexpression in cervical cancer tissues associated with tumor invasion depth and LNM^55^. Finally, LUNAR1 lncRNA expression was elevated in CRC tissues and cells, stimulating CRC progression through modulating miR‑495‑3p/MYCBP pathway^56^.

It is worth mentioning, that few studies have been carried out to explore peripheral blood circulatory lncRNAs SNHG3 or LUNAR1 expression in CRC patients’ and their relation to the disease clinical and pathological presentation. *Moreover, the possible involvement or interplay of both SNHG3 and LUNAR1, as mediators of Notch signaling, during CRC development or progression warrants more investigation. Therefore, the current study was carried out to explore their expression levels in the Egyptian CRC patients’ cohort as well as set their expression base-line, being compared to apparently healthy control subjects’ levels.*

In our study, findings revealed lncRNA SNHG3 expression level was considerably higher in CRC patients’ blood than in the healthy controls. In accordance to the mentioned prior studies, an increased SNHG3 expression was closely correlated to poorer clinico-pathological profile of our CRC patients’. Zhang Y et al. showed an increased SNHG3 fold-change expression in CRC patients’ tissues being associated with advanced malignant stage, positive LNM, and worse overall survival^57^. Similarly, our study showed blood SNHG3 lncRNA fold-change expression was significantly associated with advanced CRC stages (III, IV), and worse CRC clinico-pathological features, therefore, SNHG3 might be utilized to distinguish advanced stage CRC patients from early-stage ones, for patients’ stratification or sub-classification for further disease follow-up and monitoring for different treatment options (utilizing ncRNAs for precision).

Previous studies have demonstrated a positive correlation between lncRNA SNHG3 upregulation and tumor size, LNM, tumor stage, portal vein tumor thrombosis, distant metastasis, and recurrence in hepatocellular carcinoma tissues compared to normal tissues^58,59^. In-line to these findings, the lncRNA SNHG3 increased expression was correlated to larger tumor size, deeper tumor invasion T3-T4, positive vascular invasion as well as LNM in CRC patients’ cohort examined, proved more by the multiple regression analysis results being dependent variables. However, there was no significant correlation between SNHG3 elevated expression and tumor differentiation, mucinous tumors, or the presence of the histological signet ring cell characteristic.

Likewise, we identified lncRNA LUNAR1 expression level was increased in CRC patients’ blood compared to the healthy controls. But, in our study LUNAR1 didn’t display difference in expression among early and advanced CRC stages. Zhang Z et al. showed that an increased LUNAR1 expression level in CRC tissues as result of Notch1 cascade stimulation was significantly linked to CRC differentiation status, tumour invasion depth, and LNM, but there was no substantial association between LUNAR1 and CRC tumour size or distant metastases^37^. Comparably, LUNAR1, in the current study, enhanced expression levels were associated with more tumor invasion depth as seen in T3-T4.

Afterwards, in order to assess the utility of these two lncRNAs, ROC curves were computed. Previously, SNHG3, potentially, served as a diagnostic molecular marker in ovarian cancer with highly significant AUC^38^. As well as a published systemic review and meta-analysis claimed SNHG3 would serve as a diagnostic molecular biomarker in different types of cancer^60^. Additionally, in DLBCL^36^ and in cervical cancer^55^ LUNAR1 served as potential screening marker, which is the case in our study, if LUNAR1 expression being combined with CEA or SNHG3, as SNHG3 + LUNAR1 as combined model have better AUC, SN and SP than CEA and/or CA19.9. Additionally, the ROC curve analysis proved that by integrating CEA and CA19.9 with these lncRNAs yields AUC, SN, and SP better than those of CEA and/or CA19.9 alone. These findings imply that SNHG3 and LUNAR1 have a significantly greater ability to identify CRC than the current traditional TMs, but enhance their performance and positivity, for disease progression monitoring, in the instances of false-negative TMs cases with imaging-proved CRC cases, and finally the bases for precision treatment.

Positive correlation between SNHG3 and LUNAR1 expressions in CRC patients’ blood is an asset in the current study, comes in line with the in silico search result, with their expression levels influencing each other via tumor size and vascular invasion as independent variables’.

***Utilizing the Notch-related oncogenic lncRNAs SNHG3 and LUNAR1, as potential peripheral blood-based disease-molecular indicators, useful for CRC risk assessment and CRC patients’ sub-classification for better prognosis and/or precise disease monitoring, a step toward ncRNA-based-precision.***

Recommendation. To analyze the precise mechanism(s) involving lncRNAs SNHG3 and LUNAR1 in relation to the possible potential regulators of Notch-signaling in CRC tissues, extracted via in silico analysis prediction where IGF1R, IRS, EGF, NOTCH 1 proteins and genes expression levels to be measured, as per STRING interactions, PathCards, and GenesLikeMe bioinformatics databases.

https://string-db.org/cgi/network?taskId=bZ7mNRqKITtS&sessionId=bb3mRWhtNINO&forward_to_new_taskId_if_necessary=1&limit=0&additional_network_nodes=0 and https://pathcards.genecards.org/Card/notch_signaling_pathways?queryString=notch1

And https://glm.genecards.org/#results

Per, identification of the in silico functional enrichment analysis of Notch-signaling related genes, diseases, networks, drugs, and pathways identified the hydrolase drug **Crenigacestat** and the monoclonal antibody **Brontictuzumab**, as NOTCH-medications as well as **tamoxifen** and **sorafenib** to target SNHG3, but no drug treatment was retrieved for LUNAR1, yet. Per, LUNAR1 integrates IGF1R functional activity within, therefore, targeting *IGF1R* would be beneficial against LUNAR1. In silico STRING search identified **carvidolol, gliclazide, dalteparin, minocycline, acetylsalicylic acid, isoprenaline, caffeine, Dasatinib, Bosutinib, and Ponatinib** as such indirect treatments.

Second, identification of the pro-anti-inflammatory mediators or cytokines (granzyme, perforin system, and serpinB9) or oxidative status as well as adipokines or adipochemokines levels and/or their polymorphism in relation to LUNAR1 and SNGH3 in the clinical setting in CRC patients cohort with or without D.M or CVD.

## Conclusions

Our findings pointed out to the NOTCH-related lncRNAs SNHG3 and LUNAR1 blood fold-change expression levels were considerably higher in CRC patients and exhibited considerable clinical utility for CRC dynamic surveillance. Both lncRNAs might constitute prospective and potential non-invasive bio-molecular markers for CRC risk assessment and/or disease progression monitoring.

Being correlated with poorer patient’s clinical features, SNHG3 and LUNAR1 might constitute putative targets or candidates for future validation, unless functional assays are included, after identifying their down-stream target proteins or genes to block, as suggested by bioinformatics analysis (pending experimental verification; limitation).

A sustainability perspective ongioing research addressing the role of SNHG3 or LUNAR1 or their combined model in an independent cohort, and in normal and cancer tissue samples, with blood samples, for more validation are instigated ahead. Limitations to acknolewdge are being a single-center design and lack of functional assays (future recommendation) and to compare the expression levels of SNHG3 and LUNAR1 according to the treatment response in CRC patients. Where, changes in these lncRNAs expression during induction chemotherapy and palliative chemotherapy would augment the current study findings.

## Supplementary Information

Below is the link to the electronic supplementary material.

Supplementary Material 1.

## Data Availability

“Data is provided within the manuscript or supplementary information files”.

## Abbreviations

AJCC: American joint committee on cancer
ALT: Alanine aminotransferase
AUC: Area under the ROC curve
AST: Aspartate aminotransferase
ASUH: Ain Shams University Hospitals
BMI: Body mass index
CA19.9: Carbohydrate antigen 19.9
CEA: Carcinoembryonic antigen
CRC: Colorectal cancer
cDNA: Complementary DNA
Ct: Cycle threshold
DM: Diabetes mellitus
DLBCL: Diffuse large B-cell lymphoma
EMT: Epithelial-mesenchymal transition
GSEA: Gene set enrichment analysis
Hgb: Hemoglobin
GAPDH: Human glyceraldehyde 3-phosphate dehydrogenase
HTN: Hypertension
HGNC: HUGO Gene Nomenclature Committee
Has: Human homo sapiens
I.C: Informed consent
IGF-1R: Insulin-like growth factor 1 receptor
IQR: Interquartile range
KEGG: Kyoto Encyclopedia of Genes and Genomes
LncRNAs: Long non coding RNAs
LUNAR1: Leukemia-associated non-coding IGF1R activator RNA 1
LFTs: Liver function tests
LNM: Lymph node metastasis
miRNAs: MicroRNAs
LRs: Negative likelihood ratios
NCBI: National Center for Biotechnology Information
ncRNAs: Non-protein coding RNAs’
NPVs: Negative predictive values
PLR: Platelet-to-lymphocytes ratio
PPVs: Positive predictive values
PT: Prothrombin time
qRT-PCR: Quantitative real-time polymerase chain reaction
REC: Review board Research Ethical Committee
ROC: Receiver operating characteristic
SNs: Sensitivities
SNHG3: Small nucleolar RNA host gene 3
SPs: Specificities
TMs: Tumor markers
TNM: Tumor, node, metastasis

## References

[1] Bray, F. et al. Global cancer statistics 2018: GLOBOCAN estimates of incidence and mortality worldwide for 36 cancers in 185 countries. *CA Cancer J. Clin.* **68**(6), 394–424 (2018). 30207593 10.3322/caac.21492

[2] Sung, H. et al. Global Cancer Statistics 2020: GLOBOCAN estimates of Incidence and mortality worldwide for 36 cancers in 185 countries. *CA Cancer J. Clin.* **71**(3), 209–249 (2021). 33538338 10.3322/caac.21660

[3] Arnold, M. et al. Global patterns and trends in colorectal cancer incidence and mortality. *Gut* **66**, 683–691 (2017). 26818619 10.1136/gutjnl-2015-310912

[4] Tang, X. J., Wang, W. & Hann, S. S. Interactions among lncRNAs, miRNAs and mRNA in colorectal cancer. *Biochimie* **163**, 58–72 (2019). 31082429 10.1016/j.biochi.2019.05.010

[5] Weizman, A. V. & Nguyen, G. C. Colon cancer screening in 2010: An up-date. *Minerva Gastroenterol. Dietol.* **56**(2), 181–188 (2010). 20485255

[6] Sheng, H. et al. Adenocarcinoma with mixed subtypes is a rare but aggressive histologic subtype in colorectal cancer. *BMC Cancer* **19**(1), 1–11 (2019). 31703713 10.1186/s12885-019-6245-5 PMC6842229

[7] Pesta, M. et al. Plasma microRNA levels combined with CEA and CA19-9 in the follow-up of colorectal cancer patients. *Cancers* **11**(6), 864 (2019). 31234350 10.3390/cancers11060864 PMC6627112

[8] Lech, G., Słotwiński, R., Słodkowski, M. & Krasnodębski, I. W. Colorectal cancer tumour markers and biomarkers: Recent therapeutic advances. *World J Gastroenterol.* **22**(5), 1745 (2016). 26855534 10.3748/wjg.v22.i5.1745 PMC4724606

[9] Sokolov, D. et al. Differential signaling pathways in medulloblastoma: Nano-biomedicine targeting non-coding epigenetics to improve current and future therapeutics. *Curr. Pharm. Des.* **30**(1), 31–47. 10.2174/0113816128277350231219062154 (2024). 38151840 10.2174/0113816128277350231219062154

[10] Eissa, S. et al. Diagnostic value of urinary molecular markers in bladder cancer. *Anticancer Res.* **23**(5B), 4347–4355 (2003). 14666650

[11] Sheriff, S. et al. A scoping review of factors influencing the implementation of liquid biopsy for cancer care. *J. Exp. Clin. Cancer Res.* **44**(1), 50 (2025). 39934875 10.1186/s13046-025-03322-w PMC11817833

[12] Crocetto, F. et al. Liquid biopsy: Current advancements in clinical practice for bladder cancer. *J. Liquid Biopsy*10.1016/j.jlb.2025.100310 (2025). 10.1016/j.jlb.2025.100310 PMC12281373 40698358

[13] Eitan LA, Khair IY, Alahmad S. Drug metabolizing enzymes: An exclusive guide into latest research in pharmaco-genetic dynamics in Arab countries. Current Drug Metabol. 2024. 10.2174/0113892002323910240924145310 39377381

[14] El-Mesallamy, H. O., Hamdy, N. M. & Sallam, A.-A.M. Effect of obesity and glycemic control on serum lipocalins and insulin-like growth factor axis in type 2 diabetic patients. *Acta Diabetol.* **50**(5), 679–685. 10.1007/s00592-012-0373-6 (2013). 22307870 10.1007/s00592-012-0373-6

[15] Jahani, M. M., Mashayekhi, P., Omrani, M. D. & Meibody, A. A. Efficacy of liquid biopsy for genetic mutations determination in non-small cell lung cancer: A systematic review on literatures. *BMC Cancer* **25**(1), 433. 10.1186/s12885-025-13786-w (2025). 40065316 10.1186/s12885-025-13786-w PMC11895383

[16] Radwan, S. M., Hamdy, N. M., Hegab, H. M. & El-Mesallamy, H. O. Beclin-1 and hypoxia-inducible factor-1α genes expression: Potential biomarkers in acute leukemia patients. *Cancer Biomark.* **16**(4), 619–626. 10.3233/CBM-160603 (2016). 27002764 10.3233/CBM-160603 PMC13016510

[17] Alrumaihi, F. Role of liquid biopsy for early detection, prognosis, and therapeutic monitoring of hepatocellular carcinoma. *Diagnostics* **15**(13), 1655 (2025). 40647654 10.3390/diagnostics15131655 PMC12249386

[18] El-Mesallamy, H. O., Hamdy, N. M., Zaghloul, A. S. & Sallam, A. M. Clinical value of circulating lipocalins and insulin-like growth factor axis in pancreatic cancer diagnosis. *Pancreas* **42**(1), 149–154. 10.1097/MPA.0b013e3182550d9d (2013). 22617715 10.1097/MPA.0b013e3182550d9d

[19] El-Sheikh, N. M., Abulsoud, A. I., Fawzy, A., Wasfey, E. F. & Hamdy, N. M. LncRNA NNT-AS1/hsa-miR-485–5p/HSP90 axis in-silico and clinical prospect correlated-to histologic grades-based CRC stratification: A step toward ncRNA Precision. *Pathol. Res. Pract.* **247**, 154570. 10.1016/j.prp.2023.154570 (2023). 10.1016/j.prp.2023.154570 37244051

[20] Eldash, S., Sanad, E. F., Nada, D. & Hamdy, N. M. The intergenic type LncRNA (LINC RNA) faces in cancer with in silico scope and a directed lens to LINC00511: A step toward ncRNA precision. *Non-Coding RNA.* **9**(5), 58 (2023). 37888204 10.3390/ncrna9050058 PMC10610215

[21] El-Mesallamy, H. O., Hamdy, N. M., Zaghloul, A. S. & Sallam, A. M. Serum retinol binding protein-4 and neutrophil gelatinase-associated lipocalin are interrelated in pancreatic cancer patients. *Scand. J. Clin. Lab. Invest.* **72**(8), 602–607. 10.3109/00365513.2012.723135 (2012). 23020231 10.3109/00365513.2012.723135

[22] El-Mesallamy, H. O., Hamdy, N. M., Ezzat, O. A. & Reda, A. M. Levels of soluble advanced glycation end product-receptors and other soluble serum markers as indicators of diabetic neuropathy in the foot. *J. Investig. Med.* **59**(8), 1233–1238. 10.2310/JIM.0b013e318231db64 (2011). 10.2130/JIM.0b013e318231db64 21941211

[23] Luo, Z. et al. Notch signaling in osteogenesis, osteoclastogenesis, and angiogenesis. *Am. J. Pathol.* **189**(8), 1495–1500 (2019). 31345466 10.1016/j.ajpath.2019.05.005 PMC6699068

[24] Lai, E. C. Notch signaling: Control of cell communication and cell fate. *Development* **131**(5), 965–973 (2004). 14973298 10.1242/dev.01074

[25] Rajendran DT, Subramaniyan B, Ganeshan M. Role of Notch signaling in colorectal cancer. In: Role of Transcription Factors in Gastrointestinal Malignancies. 2017. p. 307–14.

[26] Radtke, F. & Clevers, H. Self-renewal and cancer of the gut: Two sides of a coin. *Science* **307**(5717), 1904–1909 (2005). 15790842 10.1126/science.1104815

[27] Reicher, A., Foßelteder, J., Kwong, L. N. & Pichler, M. Crosstalk between the Notch signaling pathway and long non-coding RNAs. *Cancer Lett.* **420**, 91–96 (2018). 29409809 10.1016/j.canlet.2018.01.070

[28] Emam, O., Wasfey, E. F. & Hamdy, N. M. Notch-associated lncRNAs profiling circuiting epigenetic modification in colorectal cancer. *Cancer Cell Int.* **22**(1), 316 (2022). 36229883 10.1186/s12935-022-02736-2 PMC9558410

[29] Yang, X., Duan, B. & Zhou, X. Long non-coding RNA FOXD2-AS1 functions as a tumor promoter in colorectal cancer by regulating EMT and Notch signaling pathway. *Eur. Rev. Med. Pharmacol. Sci.* **21**(16), 3586–3591 (2017). 28925486

[30] Bian, Z. et al. Long non-coding RNA LINC00152 promotes cell proliferation, metastasis, and confers 5-FU resistance in colorectal cancer by inhibiting miR-139-5p. *Oncogenesis.* **6**(11), 395 (2017). 29180678 10.1038/s41389-017-0008-4 PMC5868057

[31] Xu, J. et al. Long noncoding RNA DSCAM-AS1 facilitates colorectal cancer cell proliferation and migration via miR-137/notch1 axis. *J. Cancer.* **11**(22), 6623–6632 (2020). 33046983 10.7150/jca.46562 PMC7545673

[32] Xu, B. et al. LncRNA SNHG3, a potential oncogene in human cancers. *Cancer Cell Int.* **20**(1), 536 (2020). 33292213 10.1186/s12935-020-01608-x PMC7640707

[33] Jiang, H., Li, X., Wang, W. & Dong, H. Long non-coding RNA SNHG3 promotes breast cancer cell proliferation and metastasis by bindin g to microRNA-154-3p and activating the notch signaling pathway. *BMC Cancer* **20**, 838 (2020). 32883233 10.1186/s12885-020-07275-5 PMC7469338

[34] Zhang, L. et al. lncRNA SNHG3 acts as oncogene in ovarian cancer through miR-139-5p and Notch1. *Oncol Lett.* **21**(2), 1 (2021). 33552243 10.3892/ol.2020.12383 PMC7798025

[35] Huang, W. et al. The long non-coding RNA SNHG3 functions as a competing endogenous RNA to promote malignant development of colorectal cancer. *Oncol Rep.* **38**(3), 1402–1410 (2017). 28731158 10.3892/or.2017.5837 PMC5549033

[36] Peng, W. & Feng, J. Long noncoding RNA LUNAR1 associates with cell proliferation and predicts a poor prognosis in diffuse large B-cell lymphoma. *Biomed. Pharmacother.* **77**, 65–71 (2016). 26796267 10.1016/j.biopha.2015.12.001

[37] Zhang, Z. et al. The novel notch-induced long noncoding RNA LUNAR1 determines the proliferation and prognosis of colorectal cancer. *Sci. Rep.* **9**(1), 1–9 (2019). 31882986 10.1038/s41598-019-56536-2 PMC6934546

[38] Liu, E. L., Zhou, Y. X., Li, J., Zhang, D. H. & Liang, F. Long-chain non-coding RNA snhg3 promotes the growth of ovarian cancer cells by targeting MIR-339-5p/TRPC3 axis. *Onco Targets Ther.* **13**, 10959 (2020). 33149611 10.2147/OTT.S249873 PMC7604867

[39] Edge SB, Compton CC. The American Joint Committee on Cancer: the 7th Edition of the AJCC Cancer Staging Manual and the Future of TNM. Ann Surg Oncol. 2010; 17(6):1471–4. 10.1245/s10434-010-0985-4 20180029

[40] Subramanian, A. et al. Gene set enrichment analysis: A knowledge-based approach for interpreting genome-wide expression profiles. *Proc. Natl. Acad. Sci.* **102**(43), 15545–15550 (2005). 16199517 10.1073/pnas.0506580102 PMC1239896

[41] Kanehisa, M., Furumichi, M., Sato, Y., Kawashima, M. & Ishiguro-Watanabe, M. KEGG for taxonomy-based analysis of pathways and genomes. *Nucleic Acids Res.* **51**(D1), D587–D592 (2023). 36300620 10.1093/nar/gkac963 PMC9825424

[42] Kanehisa, M. & Goto, S. KEGG: Kyoto encyclopedia of genes and genomes. *Nucleic Acids Res.* **28**(1), 27–30 (2000). 10592173 10.1093/nar/28.1.27 PMC102409

[43] Laufs, D., Peters, M. & Schultz, C. Data platforms for open life sciences—A systematic analysis of management instruments. *PLoS ONE* **17**(10), e0276204 (2022). 36282849 10.1371/journal.pone.0276204 PMC9595524

[44] Seal RL, Gordon SM, Lush MJ, Wright MW, Bruford EA. genenames. org: the HGNC resources in 2011. Nucleic Acids Res. 2010;39(suppl_1):D514–9. 10.1093/nar/gkq892 PMC3013772 20929869

[45] Bao, Z. et al. LncRNADisease 2.0: An updated database of long non-coding RNA-associated diseases. *Nucl. Acids Res.* **47**(D1), D1034–D1037 (2019). 30285109 10.1093/nar/gky905 PMC6324086

[46] Zhou KR, Liu S, Cai L, Bin L. ENCORI: The encyclopedia of RNA interactomes. 2020.

[47] Kanehisa, M., Furumichi, M., Tanabe, M., Sato, Y. & Morishima, K. KEGG: New perspectives on genomes, pathways, diseases and drugs. *Nucleic Acids Res.* **45**(D1), D353–D361 (2017). 27899662 10.1093/nar/gkw1092 PMC5210567

[48] Szklarczyk, D. et al. STRING v11: Protein–protein association networks with increased coverage, supporting functional discovery in genome-wide experimental datasets. *Nucleic Acids Res.* **47**(D1), D607–D613 (2019). 30476243 10.1093/nar/gky1131 PMC6323986

[49] Schmittgen, T. D. & Livak, K. J. Analyzing real-time PCR data by the comparative CT method. *Nat. Protoc.* **3**(6), 1101–1108 (2008). 18546601 10.1038/nprot.2008.73

[50] Kang Y, Zhu X, Lin Z, Zeng M, Shi P, Cao Y, et al. Compare the Diagnostic and Prognostic Value of MLR, NLR and PLR in CRC Patients. Clin Lab. 2021;67(9). 10.7754/Clin.Lab.2021.201130 34542964

[51] Tyagi, A., Sharma, A. K. & Damodaran, C. A review on notch signaling and colorectal cancer. *Cells* **9**(6), 1549 (2020). 32630477 10.3390/cells9061549 PMC7349609

[52] Shaik JP, Alanazi IO, Pathan AAK, Parine NR, Almadi MA, Azzam NA, et al. Frequent activation of notch signaling pathway in colorectal cancers and its implication in patient survival outcome. J Oncol. 2020; 2020. 10.1155/2020/6768942 PMC7085396 32211044

[53] Ma, Q. et al. LncRNA SNHG3 promotes cell proliferation and invasion through the miR-384/hepatoma-derived growth factor axis in breast cancer. *Hum Cell.* **33**(1), 232–242 (2020). 31586299 10.1007/s13577-019-00287-9

[54] Nashwa, E.-K. et al. Upregulation of leukemia-induced non-coding activator RNA (LUNAR1) predicts poor outcome in pediatric T-acute lymphoblastic leukemia. *Immunobiology* **226**(6), 152149 (2021). 34735923 10.1016/j.imbio.2021.152149

[55] Riahi, A. et al. The novel biomarker LUNAR1 overexpression in cervical cancerous tissues specimens and its association with clinicopathological properties. *Gene Reports.* **28**, 101646 (2022).

[56] Qian, J., Garg, A., Li, F., Shen, Q. & Xiao, K. LncRNA LUNAR1 accelerates colorectal cancer progression by targeting the miR-495-3p/MYCBP axis. *Int J Oncol.* **57**(5), 1157–1168 (2020). 33300052 10.3892/ijo.2020.5128 PMC7549538

[57] Zhang, Y. et al. LncRNA SNHG3 is responsible for the deterioration of colorectal carcinoma through regulating the miR-370–5p/EZH1 axis. *Eur. Rev. Med. Pharmacol. Sci.* **25**(19), 6131–6137 (2021). 34661273 10.26355/eurrev_202110_26891

[58] Zhao, Q. et al. LncRNA SNHG3 promotes hepatocellular tumorigenesis by targeting miR-326. *Tohoku. J. Exp. Med.* **249**(1), 43–56 (2019). 31548493 10.1620/tjem.249.43

[59] Zhang, T., Cao, C., Wu, D. & Liu, L. SNHG3 correlates with malignant status and poor prognosis in hepatocellular carcinoma. *Tumor. Biol.* **37**, 2379–2385 (2016). 10.1007/s13277-015-4052-4 26373735

[60] Wang, D. et al. Potential diagnostic and prognostic value of the long non-coding RNA SNHG3 in human cancers: A systematic review and meta-analysis. *Int. J. Biol. Markers.* **37**(1), 3–12 (2022). 35130083 10.1177/03936155221077121

