# Supplementary material for: Small-nucleolar RNA host gene3 (SNHG3) and leukemia-associated non-coding IGF1R activator RNA 1 (LUNAR1) correlated with CRC patients’ clinical features: a step-toward ncRNA-precision
Source: Sci Rep. 2026 Feb 26;16:7825. doi: 10.1038/s41598-026-37432-y (PMC12954111; doi:10.1038/s41598-026-37432-y)
Supplement: Supplementary file 1 — Supplementary Material 1. [file 41598_2026_37432_MOESM1_ESM.docx]

**Supplementary Table S1: LncRNAs SNHG3 and LUNAR1 expression levels and the classical tumor markers (TMs) correlation with demographic and clinicopathological features within CRC patients (n=70)**

| Characteristics | | Correlation Coefficient | LncRNAs | | TMs | |
| --- | --- | --- | --- | --- | --- | --- |
| (Unit) | |  | **SNHG3** | **LUNAR1** | **CEA** | **CA19.9** |
| Age (Years) | Spearman | | -0.06820 | 0.03186 | 0.1134 | -0.03063 |
|  | *p-value* | | NS | NS | NS | NS |
| Gender (M/F) | Point-Biserial | | -0.073 | 0.215 | 0.12 | 0.111 |
|  | *p-value* | | NS | NS | NS | NS |
| Hgb (gm/dl) | Spearman | | -0.01271 | 0.1630 | -0.01876 | 0.06296 |
|  | *p-value* | | NS | NS | NS | NS |
| Lymphocytes (x10^3^cell/µL) | Spearman | | 0.02835 | -0.09829 | 0.06437 | -0.03912 |
|  | *p-value* | | NS | NS | NS | NS |
| AST (IU/L) | Spearman | | 0.2286 | -0.05027 | -0.1983 | 0.06238 |
|  | *p-value* | | NS | NS | NS | NS |
| Urea (mg/dl) | Spearman | | -0.1079 | -0.1594 | 0.02684 | -0.008909 |
|  | *p-value* | | NS | NS | NS | NS |
| Creatinine (mg/dl) | Spearman | | -0.1195 | -0.07138 | 0.06135 | -0.01813 |
|  | *p-value* | | NS | NS | NS | NS |
| DM | Point-Biserial | | -0.038 | -0.052 | 0.069 | 0.088 |
|  | *p-value* | | NS | NS | NS | NS |
| Tumor Site (colon, rectal) | Point-Biserial | | 0.145 | -0.051 | 0.03 | 0.047 |
|  | *p-value* | | NS | NS | NS | NS |
| Mucinous | Point-Biserial | | 0.115 | -0.07 | -0.083 | -0.082 |
|  | *p-value* | | NS | NS | NS | NS |
| CRC Family History | Point-Biserial | | -0.175 | -0.088 | -0.098 | -0.122 |
|  | *p-value* | | NS | NS | NS | NS |
| Ulcerative colitis | Point-Biserial | | 0.044 | 0.213 | 0.012 | 0.061 |
|  | *p-value* | | NS | NS | NS | NS |
| HTN | Point-Biserial | | 0.056 | -0.082 | -0.065 | -0.085 |
|  | *p-value* | | NS | NS | NS | NS |

Using graphpad prism software, Spearman correlation coefficient r was used to measure the degree of association between two continuous non-parametric variables, however point-biserial correlation was used to measure the association between two variables; one continuous and the other is dichotomous using SPSS software. Statistical significance is at *p-*value <0.05. [ALT, Alanine aminotransferase; AST, Aspartate aminotransferase; CRC, Colorectal cancer; DM, Diabetes mellitus; Hgb, Hemoglobin; HTN, Hypertension; PLR, Platelet-to-lymphocyte ratio; NS, non-significant.]
